# Role of Phytochemicals in Skin Photoprotection *via* Regulation of Nrf2

**DOI:** 10.3389/fphar.2022.823881

**Published:** 2022-05-12

**Authors:** Anyamanee Chaiprasongsuk, Uraiwan Panich

**Affiliations:** ^1^ Princess Srisavangavadhana College of Medicine, Chulabhorn Royal Academy, Bangkok, Thailand; ^2^ Department of Pharmacology, Faculty of Medicine Siriraj Hospital, Mahidol University, Bangkok, Thailand

**Keywords:** phytochemicals, photoaging, Nrf2, inflammation, skin barrier dysfunction

## Abstract

Ethnopharmacological studies have become increasingly valuable in the development of botanical products and their bioactive phytochemicals as novel and effective preventive and therapeutic strategies for various diseases including skin photoaging and photodamage-related skin problems including abnormal pigmentation and inflammation. Exploring the roles of phytochemicals in mitigating ultraviolet radiation (UVR)-induced skin damage is thus of importance to offer insights into medicinal and ethnopharmacological potential for development of novel and effective photoprotective agents. UVR plays a role in the skin premature aging (or photoaging) or impaired skin integrity and function through triggering various biological responses of skin cells including apoptosis, oxidative stress, DNA damage and inflammation. In addition, melanin produced by epidermal melanocytes play a protective role against UVR-induced skin damage and therefore hyperpigmentation mediated by UV irradiation could reflect a sign of defensive response of the skin to stress. However, alteration in melanin synthesis may be implicated in skin damage, particularly in individuals with fair skin. Oxidative stress induced by UVR contributes to the process of skin aging and inflammation through the activation of related signaling pathways such as the mitogen-activated protein kinase (MAPK)/activator protein-1 (AP-1), the phosphatidylinositol 3-kinase (PI3K)/protein kinase B (Akt), the nuclear factor kappa B (NF-κB) and the signal transducer and activator of transcription (STAT) in epidermal keratinocytes and dermal fibroblasts. ROS formation induced by UVR also plays a role in regulation of melanogenesis in melanocytes *via* modulating MAPK, PI3K/Akt and the melanocortin 1 receptor (MC1R)-microphthalmia-associated transcription factor (MITF) signaling cascades. Additionally, nuclear factor erythroid 2-related factor 2 (Nrf2)-regulated antioxidant defenses can affect the major signaling pathways involved in regulation of photoaging, inflammation associated with skin barrier dysfunction and melanogenesis. This review thus highlights the roles of phytochemicals potentially acting as Nrf2 inducers in improving photoaging, inflammation and hyperpigmentation *via* regulation of cellular homeostasis involved in skin integrity and function. Taken together, understanding the role of phytochemicals targeting Nrf2 in photoprotection could provide an insight into potential development of natural products as a promising strategy to delay skin photoaging and improve skin conditions.

## Introduction

The skin is the largest organ in the body, and one of its main functions is to protect the body from environmental stressors including ultraviolet radiation (UVR), which can result in dermatological disorders, such as skin premature aging, abnormal pigmentation and inflammatory reactions. UVR has been generally known to have both beneficial and detrimental effects on human health. While UVR plays a role in natural synthesis of vitamin D, melanin, and various peptides in the skin that have positive biological implications (Slominski et al., 2000; Lin et al., 2016), excessive exposure to UVR can lead to acute and chronic adverse effects on the health of skin and is involved in the pathogenesis of premature aging (or photoaging) and increased risk of photocarcinogenesis. In addition, UVR is accepted as human carcinogen through oxidative mechanisms accountable for increased risk of developing skin cancers including non-melanoma and melanoma skin cancers (Tran et al., 2008). The incidence of skin cancer has dramatically risen in particular among fair-skinned populations, primarily due to lifestyle changes and increased recreational exposure to UVR including outdoor activities and sunbathing for cosmetic purposes (Narayanan et al., 2010). Both male and female independent of age are affected by dermatological concerns and increasingly interested in rejuvenation and skin cancer prevention. While the use of sunscreens is recommended in order to minimize the risk of photoaging and other UV-related skin disorders, sunscreen alone does not provide sufficient protection against deleterious effects of UVR. Oxidative stress plays a crucial role in UVR-induced photodamage *via* mediating multiple biological responses including apoptosis, DNA damage, mitochondrial dysfunction, inflammation, abnormal pigmentation and upregulation of matrix metalloproteinases (MMPs) (such as MMP-1) in related skin cell types including keratinocytes, fibroblasts and melanocytes (Liebel et al., 2012; Denat et al., 2014; Silva et al., 2017; Lohakul et al., 2021b). Therefore, cellular and molecular regulation of antioxidant defenses to combat oxidative stress and promote redox balance could be a potential therapeutic and preventive strategies for photodamaged skin.

Nuclear factor erythroid 2-related factor 2 (Nrf2) is an important transcription factor controlling antioxidant responses in various tissues including the skin and plays a major role in cytoprotection against chemical and environmental insults including UVR (Ikehata and Yamamoto, 2018). Targeting Nrf2 could improve photoaging, wound repair and dyspigmentation as well as prevent photocarcinogenesis *via* regulation of cellular homeostasis involved in skin integrity and function (Saw et al., 2011; Gegotek and Skrzydlewska, 2015). Phytochemicals, which are ubiquitously present in plant-based diets and are active ingredients in several botanical drugs, have thus gained remarkable attention as promising candidates for effective photoprotective agents due to their abilities to activate Nrf2 signaling-regulated redox balance and subsequently maintain cellular homeostasis involved in skin integrity and function. Much attention has been focused on the role of dietary polyphenols in the repair of photodamaged skin and prevention of solar-induced skin diseases (Surh and Na, 2008; Saw et al., 2011; Dunaway et al., 2018). In this review, we provide an overview of the promising roles of phytochemicals in mitigating UVR-induced skin damage via regulation of Nrf2-mediated antioxidant response to offer an insight into ethnopharmacological potential for development of novel and effective anti-photodamaging agents.

### The Role of UVR-Induced Oxidative Stress in Skin Photodamage

The skin is a primary target of oxidative stress because it is constantly exposed to environment including UVR, which induces reactive oxygen species (ROS) generation in the skin. It has been well accepted that both UVA and UVB rays play a significant role in the premature aging and photodamage of the skin through various mechanisms involving oxidative stress (Lephart, 2016; Gegotek et al., 2017). While UVB has biological impact on the skin primarily by causing direct damage to DNA and inflammation (Halliday and Lyons, 2008), UVA accounts for skin photodamage by generating various types of ROS, such as superoxide anion radical (O_2_^•-^), singlet oxygen (^1^ΔgO_2_) and hydrogen peroxide (H_2_O_2_). ROS can interact with biomolecules and interfere with cell signaling, affecting cell survival and function of the skin cells (Dunaway et al., 2018). Considerable studies have reported that UVA exposure significantly led to ROS accumulation responsible for oxidative damage to biomolecules including DNA (O'Donovan et al., 2005), lipid (Dissemond et al., 2003) and protein in the skin cells including fibroblasts, keratinocytes (Brem et al., 2017) and melanocytes. Oxidative damage mediated by both UVA and UVB is associated with apoptosis and necrosis of the skin cells associated with sunburn reaction and photoaging process (Didier et al., 2001; Suschek et al., 2001; Kawachi et al., 2008; Parrado et al., 2016).

### Skin Photoaging

Photoaging is characterized by epidermal thickness, termed hyperkeratosis, due to increased keratinocyte hyperproliferation as well as degradation or degeneration and disorganization of collagen fibers caused by upregulation of MMPs (Quan et al., 2009; Pittayapruek et al., 2016). In addition, dysregulated proliferation of transformed neoplastic keratinocytes or actinic keratosis is the key event in the progression from photoaged skin to squamous cell carcinoma (Berman and Cockerell, 2013). UVR (both UVA and UVB) is well accepted to play a vital role in photoaging *via* several mechanisms including DNA damage, oxidative stress, apoptosis, senescence, inflammation, immunomodulation (Rijken and Bruijnzeel-Koomen, 2011; Brand et al., 2017) and degradation and/or remodeling of the extracellular matrix (ECM) (Bosch et al., 2015). Generally, the characteristic hallmarks of photoaged skin are alterations in the ECM including accumulation of disorganized elastin fibers and depletion of collagens, the main structural proteins of the dermal connective tissues. Both UVA and UVB radiation can induce hyperkeratosis and several types of MMPs (including MMP-1 or collagenase) in mouse models of photoaging (Chaiprasongsuk et al., 2017; Misawa et al., 2017). Several *in vitro* and *in vivo* studies have reported that UV irradiation stimulates expression of MMP-1, MMP-3 and MMP-9, which are the major UV-inducible collagenolytic enzymes, regulated at the transcriptional level (Afaq et al., 2009; Quan et al., 2009; Pittayapruek et al., 2016). MMPs are co-expressed in response to various stimuli including oxidative insults, inflammatory cytokines and growth factors (Greenlee et al., 2007; Lee et al., 2021). MMPs are suggested to be downstream targets within signaling pathways of upstream response genes, which encode several signaling proteins that activate different transcription factors capable of binding the promoters of MMP genes. The key transcription-binding sites involved in the regulation of MMP genes include the activator protein-1 (AP-1) site, the nuclear factor kappa B (NF-κB) site and the signal transducer and activator of transcription (STAT) site (Fanjul-Fernandez et al., 2010). In addition, MMPs can be co-regulated because they share several transcription-binding sites in their promoter sequences. NF-κB and AP-1 are the transcription factors that can bind the promoters of MMP-1, 3 and 9 (Watanabe et al., 2004). The AP-1 transcription complex, a family of dimeric transcription factors composed of members of the Jun and Fos family proteins, is the main transcription factor regulating MMP-1 gene (Angel et al., 2001). In general, the c-Jun and c-Fos genes are activated rapidly and transiently in response to stimuli and are thus considered immediate-early response genes. Binding of heterodimer complexes of c-Jun with c-Fos to the AP-1 site, which is specific DNA sequences (5′-TGAG/CTCA-3′), termed TREs (TPA (tetradecanoylphorbol-12-Acetate)-response elements), is responsible for transactivation of AP-1 that regulates MMP-1, 3 and 9 expressions (Mackay et al., 1992; Watanabe et al., 2004). Both c-Jun and c-Fos are controlled by mitogen-activated protein kinase (MAPK) signaling pathways which are stimulated by extracellular stimuli including growth factors and cytokines as well as environmental stimuli including UVR. Three distinct types of MAPKs, ERK (extracellular signal-regulated kinase), JNK (c-Jun NH_2_-terminal kinase) and p38 MAPK, differentially affect AP-1 activity in response to various stimuli (Karin, 1995). The ERKs generally are triggered by growth factors and hormones as well as JNK and p38 MAPK are activated by environmental stresses including UVR and pro-inflammatory mediators, such as tumor necrosis factor (Chang and Karin, 2001; Silvers et al., 2003; Whitmarsh, 2007). UVR is suggested to primarily cause the greatest increases in JNK activity. Upon exposure to some stimuli, phosphorylation of c-Fos in the AP-1 complex at two C-terminal sites (Ser362 and Ser374) by MAPKs, in particular ERK, is required for transactivation at the specific AP-1 site (McBride and Nemer, 1998). In addition, c-Jun is activated and stabilized by JNK- and p38-catalyzed phosphorylation at the NH_2_-terminal sites (Ser63 and Ser73) located within transactivation domain of c-Jun. p38 MAPK indirectly activates AP-1 by phosphorylating other transcription factors such as AP-1 family proteins ATF2 (the activating transcription factor) forming a heterodimer with c-Jun, which then binds to the promoter elements in the c-Jun gene and regulates its transcription, leading to the subsequent upregulation of c-Jun expression and synthesis (Pramanik et al., 2003).

ROS participates in the photoaging process through several mechanisms including DNA damage, apoptosis, upstream modulation of MAPK/AP-1, NF-κB and JAK (janus kinase)-STAT signaling cascades, activation of cytokine and growth factor receptors and immune reaction of melanocytes and keratinocytes. Upregulation of MAPK/AP-1 signaling results in induction of transcription and production of MMPs (such as collagenase-1 (MMP-1), stromelysin-1 (MMP-3), and gelantinase A (MMP-2), that subsequently degrade ECM including collagen and elastin as well as suppress the collagen synthesis in the dermal fibroblasts. Furthermore, keratinocytes play an indirect role in photoaging through secreting paracrine factors, which stimulate the signaling cascades-mediated upregulation of MMPs in dermal fibroblasts. Several studies including ours suggested that ROS formation is involved in the molecular mechanisms of photoaging *via* activating MAPK/AP-1 signaling pathway, resulting in both upregulation of MMPs and downregulation of procollagen I production (Li et al., 2019). ROS induced by UVR is implicated in MAPKs-dependent activation of AP-1 signaling, leading to upregulation of various MMPs including MMP-1, MMP-3 and MMP-9 in both keratinocytes and fibroblasts (Rittie and Fisher, 2002; Pittayapruek et al., 2016). Our *in vitro* and *in vivo* studies demonstrated the role of ROS induced by UVA exposure in upregulation of MMP-1 through activation of MAPK/AP-1 signaling pathway in keratinocytes and mouse skin. In addition, UVB has been shown to trigger MMP-1 and MMP-3 expressions through ROS generation and MAPK/AP-1 activation in irradiated keratinocytes and fibroblasts (Kim et al., 2013; Kim et al., 2015; Lu et al., 2016). In addition, UVB radiation is suggested to induce ROS formation, leading to increased MMP-9 activity and expression in mouse embryonic fibroblasts and HaCaT keratinocytes (Chang et al., 2017; Ma et al., 2018). UVB-induced oxidative stress was observed to activate MAPK signaling in association with increased expression of MMP-9 in UVB-exposed dermal fibroblasts (Gunaseelan et al., 2017). Moreover, ROS was found to be involved in activation of p38 MAPK and induction of MMP-9 expression in UVB-exposed HaCaT keratinocytes (Li et al., 2017b) and mouse dermis (Li et al., 2017b). In addition to collagen and elastin degradation by MMPs, UVB plays a role in a reduction of procollagen type I synthesis through activating AP-1-mediated downregulation of transforming growth factor beta (TGF-β) signaling (Pittayapruek et al., 2016; Gao et al., 2018a).

It should also be taken into account that photoaging of the skin is a complex multifactorial process. Apart from ROS/MAPK/AP-1 signaling cascades, UVR which can activate various cell surface receptors can stimulate downstream signaling pathways that control other different transcription factors regulating expression of many genes involved in photoaging process. Eventually, it is important to inhibit ECM degradation that leads to solar scar, a process taking place with each exposure to UV even at low doses. Since ROS serve as important second messengers, which act upstream of MAPK/AP-1 signaling-mediated induction of MMPs and reduction of procollagen implicated in pathogenesis of photoaging, controlling ROS homeostasis could represent promising pharmacological and molecular approaches to impede photoaging.

### Skin Inflammation and Skin Barrier Dysfunction

The skin barrier dysfunction and oxidative stress are suggested to play a role in the development of chronic inflammatory skin conditions (e.g., dermatitis) and alteration of wound healing process (Wikramanayake et al., 2014). The accumulation and alteration of external stimuli exposures result in a compromised barrier function of the skin through cutaneous inflammation and the imbalance of skin homeostasis (Egawa and Kabashima, 2018). Previous studies have demonstrated that UVB irradiation has a negative impact on epidermal morphology and barrier function by increasing stratum corneum (SC) thickness, causing changes in SC lipids and stimulating transepidermal water loss (Biniek et al., 2012). ROS generation induced by UVR plays a role in epidermal barrier dysfunction through oxidative damage to proteins and lipids, leading to alteration of tissue structure (Rinnerthaler et al., 2015). Regulation of Nrf2 activity has also been proposed to offer a potential strategy to improve skin barrier integrity by mitigating UVR-induced damage of keratinocytes and modulating inflammatory responses of the skin. Downregulation of Nrf2 signaling was shown to be involved in UVB-induced upregulation of pro-inflammatory mediators such as tumor necrosis factor alpha (TNF-α), cyclooxygenase-2 (COX-2), interleukin-6 (IL-6), interleukin-1 beta (IL-1β), and interleukin-8 (IL-8) in keratinocyte HaCaT cells (Park et al., 2021). A previous study using a mouse model of UVB-induced photodamage revealed that basal activity of Nrf2 in keratinocytes of normal skin is vital for improvement of skin barrier integrity and for prevention of skin carcinogenesis. UVB-mediated apoptosis of epidermal cells was involved in impaired skin integrity and activation of Nrf2 was observed to protect against UVB-induced apoptosis of basal keratinocytes in a paracrine, glutathione (GSH)/cysteine-dependent manner. Furthermore, enhanced levels of Nrf2-dependent genes in all layers of epidermis in response to UVB exposure were involved in the suppression of apoptosis *in vivo* (Schafer et al., 2010). The connection between the Nrf2 and antioxidant response element (ARE) system proved the protective pathways of skin inflammation via the regulation of the inflammatory factors (Saha et al., 2020). The *NFE2L2* gene encoding for Nrf2 contains ARE-like sequences, providing a positive feedback mechanism to amplify antioxidant and anti-inflammatory signaling such as glutathione S-transferase (GST), NAD(P)H quinone oxidoreductase-1 (NQO-1), heme oxygenase-1 (HO-1) (Nguyen et al., 2009; Luo et al., 2018). Nrf2 has been suggested to play a role in modulating several signaling pathways involved in the inflammatory responses include NF-κB, MAPK, and JAK-STAT. Previous studies have reported the crosstalk between Nrf2 and NF-κB pathway. Nrf2 negatively regulated the NF-κB signaling pathway and proinflammatory cytokine production by inhibiting oxidative stress-induced NF-κB and preventing the IκB-α (NF-κB inhibitor) proteasomal degradation (Ma, 2013; Saha et al., 2020). In addition, several proinflammatory cytokines (e.g., IL-6, TNF-α and IL-1β), growth factors (e.g., epidermal growth factor (EGF), fibroblast growth factor, keratinocyte growth factor (KGF) and vascular endothelial growth factor (VEGF) and MMPs (e.g., MMP-2 and MMP-9) play a role in wound repair consisting of a series of multiple stages including inflammation, proliferation and remodeling (Shao et al., 2019; Suntar et al., 2021). Activation of Nrf2 in response to ROS production in inflamed tissues is thus suggested to play a role in promoting wound healing and regulating repair-related inflammation. Moreover, Nrf2 transcripts several genes encoding skin barrier structural and functional components including the keratins (KRT), the cornified envelope family members, small proline rich proteins, secretory leukocyte protease inhibitor, and the EGF family member epigen (Rojo de la Vega et al., 2017). In addition to Nrf2, STAT3 (in cell proliferation and differentiation), Smad proteins (in collagen production) and Forkhead box protein N1 (FOXN1) (in re-epithelization) are important transcriptional regulators involved in the process of wound repair. Furthermore, without involvement of inflammatory cells, upregulation of Nrf2 activity and its target antioxidant NQO-1 or HO-1 was demonstrated to promote the migration of corneal epithelial cells during wound repair *in vitro* and *in vivo* (Hayashi et al., 2013). In response to ROS produced in the early phase of wound repair, upregulation of Nrf2 as a target of KGF in keratinocytes is involved in the healing process in association with modulating proinflammatory cytokine IL-1, IL-6, and TNF-α and TGF-β1 and VEGF *in vitro* and *in vivo* (Braun et al., 2002). Nevertheless, the regulatory role of Nrf2 in epidermal homeostasis is complex and needs further clarification as prolonged activation of Nrf2 in keratinocytes could interfere skin homeostasis. Previous *in vitro* and *in vivo* studies demonstrated that increased activity of Nrf2 in keratinocytes resulted in epithelial abnormalities, altered epidermal barrier and development of hyperkeratosis (Kypriotou et al., 2012; Schafer et al., 2012).

### Skin Hyperpigmentation

While melanin plays a crucial role in protecting the skin against harmful effects of UVR, excessive production of melanin could be detrimental because melanin precursors and intermediate metabolites produced during melanogenesis in response to UVR exert phototoxic properties (Schmitz et al., 1995). Whereas hyperpigmentation mediated by UV irradiation could reflect a sign of defensive response of the skin to stress, alteration in melanin synthesis may be implicated in skin damage, particularly in individuals with fair skin. UVR-dependent elevated melanogenesis has been suggested to be biologically harmful, genotoxic and contributed to development of melanoma skin cancer, especially in lightly pigmented individuals. The incidence of skin cancer has dramatically risen in particular among fair-skinned populations, primarily due to lifestyle changes and increased recreational exposure to UVR including outdoor activities and sunbathing for cosmetic purposes (Narayanan et al., 2010; D'Orazio et al., 2013; Watson et al., 2016). Furthermore, the growth of skin fairness products is dramatic particularly in Asia and Africa, although the use of skin bleaching products is associated with adverse side effects (Shroff et al., 2017). Thus, there is a need to develop effective and safe strategies for improvement of skin dyspigmentation or uneven complexion. Melanogenesis in melanocytes is a complex biosynthetic process involving the tyrosinase-catalyzed oxidation of tyrosine. Two main types of melanin, pheomelanin and eumelanin, are found in human skin and hair. Eumelanin is the brown/black insoluble pigment, characterizing dark phenotypes, and pheomelanin is the red/yellow, sulfur-containing pigment, predominating in red-haired individuals (Slominski et al., 2004). Eumelanin functions as a UV absorbent and subsequently has photoprotective action. Pheomelanin is photolabile and can produce ROS as by-products that lead to further DNA damage and is thus suggested to be carcinogenic following UVR (Brenner and Hearing, 2008).

Tyrosinase, a copper-containing membrane-bound located in melanosomes, catalyzes hydroxylation of L-tyrosine to L-DOPA, which is the first and the rate-limiting step of melanogenesis for both eumelanin and pheomelanin. In addition to tyrosinase, crucial enzymes involved in eumelanin synthesis include tyrosinase related proteins (TRP-1) and dopachrome tautomerase (DCT or TRP-2). Pheomelanin is produced *via* benzothiazine intermediates deriving from the oxidative polymerization of cysteinyl dopa derivatives generated through the condensation of the cysteine or GSH with the dopaquinone (Lu et al., 2021). Environmental stimuli (e.g., UVR and drugs), endogenous factors (e.g., hormone and mediators) and genetic factors can influence melanogenesis regulated by tyrosinase *via* various signal pathways, primarily the melanocortin 1 receptor (MC1R)-microphthalmia-associated transcription factor (MITF) signaling. MC1R is a G protein-coupled receptor that controls the quantity and quality of melanin synthesized in melanocytes. Important agonists of MC1-R acting as the main intrinsic regulator of pigmentation are peptide hormones and neuropeptides including stimulating hormone (α-MSH), endothelin-1 (ET-1) and adrenocorticotropic hormone (ACTH), which are cleavage products of proopiomelanocortin (POMC) (Lin and Fisher, 2007). The major signal transduction pathways that mediate the regulation of melanogenesis involve the binding of agonists to MC1R that trigger events inside melanocytes through raising intracellular cyclic 3′-5′-cyclic adenosine monophosphate (cAMP) and activating the adenylate cyclase enzyme, protein kinase A (PKA), leading to phosphorylation of the cAMP responsive binding element (CREB), which promotes the activation of MITF, which is the master transcription factor that regulates expression of several melanogenic genes including tyrosinase, TYRP1 and TYRP2. Moreover, upon activation of MC1R, enhanced levels of cAMP and subsequent activation of PKA were observed to activate the MAPK signaling cascades including p38, leading to activation of MITF (Smalley and Eisen, 2000). However, inhibition of MC1R in normal melanocytes and melanoma cells was observed to trigger PI3K/Akt and MAPK/ERK pathways, leading to inhibition of MITF and subsequent suppression of melanogenesis (Chae et al., 2017; Wu et al., 2018). Mechanisms underlying the role of phytochemicals in regulating pigmentation involve the direct suppression of tyrosinase activity and/or gene expression, direct scavenging of ROS, promotion of Nrf2-regulated antioxidant defense and inhibition of signaling pathways involved in inflammatory responses (Vomund et al., 2017; Boo, 2019). Nrf2 is suggested to play a role in modulating crucial signaling pathways including MAPK, PI3K/Akt and MC1R-MITF signaling cascades involved in regulation of melanin synthesis (Shin et al., 2014; Chaiprasongsuk et al., 2016). Moreover, exposure of the skin to UVR can stimulate keratinocytes to secrete hormones including ACTH, ET-1, α-MSH that bind to MC1R, activating MITF and upregulating melanogenesis-related proteins. Activation of Nrf2 has been observed to suppress the paracrine factors (such as α-MSH) derived from keratinocytes that results in downregulation of signaling pathways (including the cAMP/CREB/MITF pathway) involved in melanogenesis in melanocytes (Hseu et al., 2020; Chen SJ. et al., 2021). Therefore, application of compounds having abilities to activate Nrf2 might represent a promising approach to prevent and treat hyperpigmentation disorders.

Moreover, in response to UVR, melanogenesis acts as a shield against the harmful effect of UVR on the skin and thus approaches promoting melanin production can mitigate UVR-induced melanocyte damage. We previously demonstrated the role of Nrf2 in regulating the release of paracrine factor α-MSH by keratinocytes that influenced UVB-mediated melanocyte responses including DNA damage, oxidative stress, apoptosis and inflammation (Jeayeng et al., 2017). Several natural compounds such as flavonoids and coumarins having abilities to induce melanogenesis and restore melanocyte viability might thus be useful in the prevention and treatment of hypopigmentation disorders such as vitiligo (Niu and Aisa, 2017). Therefore, phytochemicals have been proposed to exert beneficial effects against abnormal melanogenesis *via* improving hyperpigmentation or hypopigmentation caused by disruption of melanocyte homeostasis and/or loss of functional melanocytes. While this review highlights the studies demonstrating the roles of phytochemicals in improving UVR-induced hyperpigmentation *via* Nrf2-dependent mechanisms, it should be taken into account that melanocyte biology is complex and the role of phytochemicals in regulating melanogenesis involved in maintaining the skin homeostasis needs further clarification.

### The Role of Nrf2-Regulated Antioxidant Defense Against Cutaneous Photodamage

The primary endogenous antioxidant defenses include antioxidant and detoxification enzymes such as catalase, glutamate cysteine ligase (GCL) (composed of a catalytic subunit GCLC and a modifier subunit GCLM), the rate-limiting enzyme in GSH synthesis, glutathione peroxidase (GPx), GST, HO-1, NQO-1 and superoxide dismutase (SOD) regulated by various transcription factors including Nrf2 (Surh, 2003; Hseu et al., 2012). Nrf2 is a master regulator of antioxidant and cytoprotective genes involved in the human skin adaption to the environmental insults including UVR and thus plays a beneficial role in maintenance of skin homeostasis. Activity of Nrf2 is tightly regulated by proteins including Kelch-ECH associated protein 1 (Keap 1) and proteasome degradation system and thus regulation of Nrf2-mediated antioxidant response pathway is complicated. Under homeostatic conditions, two molecules of Keap1 bound to Nrf2 is responsible for the continuous ubiquitylation and degradation of Nrf2. In response to Nrf2 activating stimuli or oxidative stress, Keap1 is oxidized at critical cysteine residues, especially Cys151, leading to dissociation of Keap1-Nrf2 that allows Nrf2 to escape from Keap1-mediated ubiquitination. Nrf2 is then translocated into the nucleus and binds to the ARE promoter, a *cis*-acting enhancer sequence located in the 5′-flanking regions of genes encoding phase II and antioxidant cytoprotective enzymes including GST, NQO-1 and GCL (Schafer et al., 2010; Liu et al., 2016; Boo, 2020b).

Nrf2 plays a vital role in maintaining redox homeostasis and cellular metabolism in skin cells involved in the skin’s structural integrity and function (Ikehata and Yamamoto, 2018). Oxidative insults, such as UVR and H_2_O_2_, and electrophilic chemicals, such as butylated hydroxyanisole and its active de-methylated metabolite *tert*-butyl hydroquinone (tBHQ); phenolic flavonoids [e.g., green tea polyphenols and epigallocatechin-3-gallate (EGCG)]; and the naturally occurring isothiocyanates including sulforaphane (SFN) and curcumin, can stimulate Nrf2 activity *via* modification of Keap1 cysteine residues, suggested as the stress sensors for Nrf2 activator (Baird and Yamamoto, 2020). The cysteine modifications result in a conformational change in the associated motif of Keap1–Nrf2 that facilitates the dissociation of Nrf2 from Keap1 and subsequently Nrf2 nuclear translocation (Kong et al., 2001). Generally, various environmental stressors including UVR lead to post-translational activation of Nrf2 through Keap1 inactivation. The upregulation of Nrf2-mediated antioxidant defense system was demonstrated *in vitro* and *in vivo* to protect the human skin from harmful effects of UVR. UVA-1-mediated lipid oxidation induces expression of antioxidant response genes, which is dependent on the redox-regulated transcription factor Nrf2 in dermal fibroblasts (Gruber et al., 2010). Exposure of keratinocytes (including primary human epidermal keratinocytes and HaCaT keratinocyte cell lines) to UVA (20 J/cm^2^) increased Nrf2 activity *via* enhancing Keap1 expression. UVA exposure led to stimulation of Nrf2 activity and its target proteins (HO-1, NQO-1, GST) in HaCaT keratinocytes and dermal fibroblasts, although Nrf2 activity was minimally affected in UVA-irradiated primary keratinocytes (Rysava et al., 2020). In fact, the regulatory role of Nrf2 in skin cell survival and function affected by UVR is complex because UVR can either upregulate or downregulate Nrf2-mediated antioxidant defense in various skin cell types. Changes in the Nrf2 activity are dynamic and dependent on types of UV ray, UVR’s intensity and time following the exposure (Chaiprasongsuk et al., 2016; Rysava et al., 2020; Rysava et al., 2021). Previous observations indicate that both UVA and UVB downregulate Nrf2 antioxidant signaling pathway in skin keratinocytes, fibroblasts and melanocytes *in vitro* and in skin tissues *in vivo*. UVB exposure led to reduced expressions of Nrf2 and its target antioxidant HO-1 proteins in HaCaT keratinocyte cells and mouse skin *in vivo* (Rodriguez-Luna et al., 2018; Rodriguez-Luna et al., 2019). UVR was observed to downregulate antioxidant and detoxifying enzymes including GST, NQO-1 and γ-GCS (γ-glutamylcysteine synthetase) in the skin cells through modulating activity of Nrf2 (Kannan and Jaiswal, 2006; Lohakul et al., 2021b). The DNA damage or modulation of signaling cascades (including MAPKs) that take place rapidly in response to UVR exposure is suggested to mediate the downregulation of Nrf2 antioxidant response pathway (Lopez-Camarillo et al., 2012). The p38 was suggested to reduce Nrf2 nuclear translocation and its transcriptional activity (Boo, 2020b). In addition, activation of Nrf2 signaling has been suggested to protect against UVR-mediated skin damage *via* several mechanisms including promotion of antioxidant and cytoprotective defense, DNA repair, anti-inflammatory signaling. Upregulation of Nrf2/HO-1 signaling accompanied with increased activities and protein levels of catalase, GPx and SOD was observed to suppress apoptosis induced by UVR [UVA (3 J/cm^2^)+UVB (90 mJ/cm^2^)] *via* activating the PI3K/Akt signaling pathway in the 3D skin model (Xian et al., 2019). Thus, understanding the role of Nrf2 in the pathogenesis of skin photodamage could give an insight into development of potential compounds having Nrf2 inducing activity for prevention and treatment of skin photodamage.

### Phytochemicals Targeting Nrf2 in Skin Photodamage: Development of Botanicals and Phytochemicals as Promising Photoprotective Agents

**FIGURE 1:**
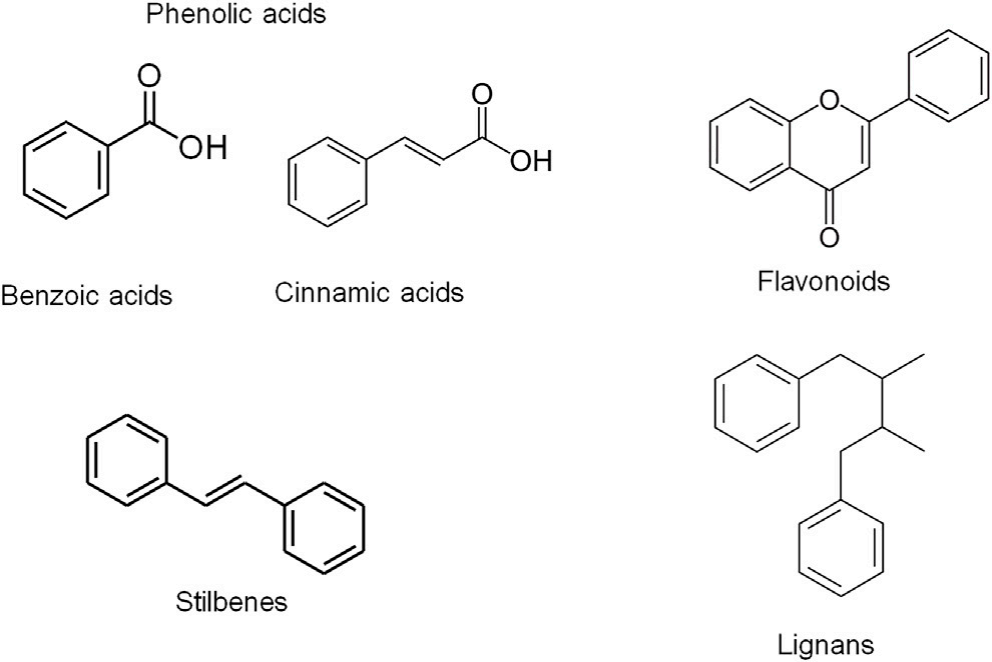
Chemical structures of the different classes of phenolic compounds including phenolic acids, flavonoids, stilbenes and lignans. Phenolic compounds are classified into different groups on the basis of the number of phenolic rings they contain and of the structural elements binding the rings to one another. They are generally divided into four classes including phenolic acids, flavonoids, stilbenes and lignans. Phenolic acids are further classified as hydroxyl benzoic and hydroxyl cinnamic acid derivatives (Johnsson, 2004; Nishiumi et al., 2011).

Several reports have highlighted the potential role of bioactive phytochemicals of plant-based diets and botanical drugs that have been used in ethnomedicine or reported in ethnopharmacological studies. The bioactive phytochemicals are naturally occurring compounds in botanical products including plants and botanical drugs which exert biological activities providing medical and nutritional benefit. These compounds exert antioxidant effects by directly scavenging ROS or by promoting the antioxidant defense system through the Nrf2-dependent pathway (L Suraweera et al., 2020). Phytochemicals include polyphenols and the non-phenolic phytochemicals. Phenolic compounds are classified into different groups on the basis of the number of phenolic rings they contain and of the structural elements binding the rings to one another. They are generally divided into four classes including phenolic acids, flavonoids, stilbenes and lignans (Figure 1). Phenolic acids are further classified as hydroxyl benzoic and hydroxyl cinnamic acid derivatives (Pandey and Rizvi, 2009). The natural sources of polyphenols include fruits (e.g., apple, berries, cherries, grapes, strawberries and pomegranate), vegetables, soybeans, cereals, tea, cocoa, soy and *Phlebodium aureum* (L.) J.Sm. The common flavonoids include catechins, quercetin, genistein, epicatechin, catechin and anthocyanins (Bosch et al., 2015). The common phenolic acids are hydroxycinnamic acids including caffeic acid and ferulic acid as well as gallic acid (also known as 3,4,5-trihydroxybenzoic acid). Gallic acid is the most abundant phenolic acid found in plant-based diets (Hano and Tungmunnithum, 2020). For non-flavonoid phenolics, the most widely studied stilbene is resveratrol. The commonly studied non-phenolic phytochemicals include carotenoids, caffeine and sulforaphane (SFN) (Bosch et al., 2015).

**FIGURE 2:**
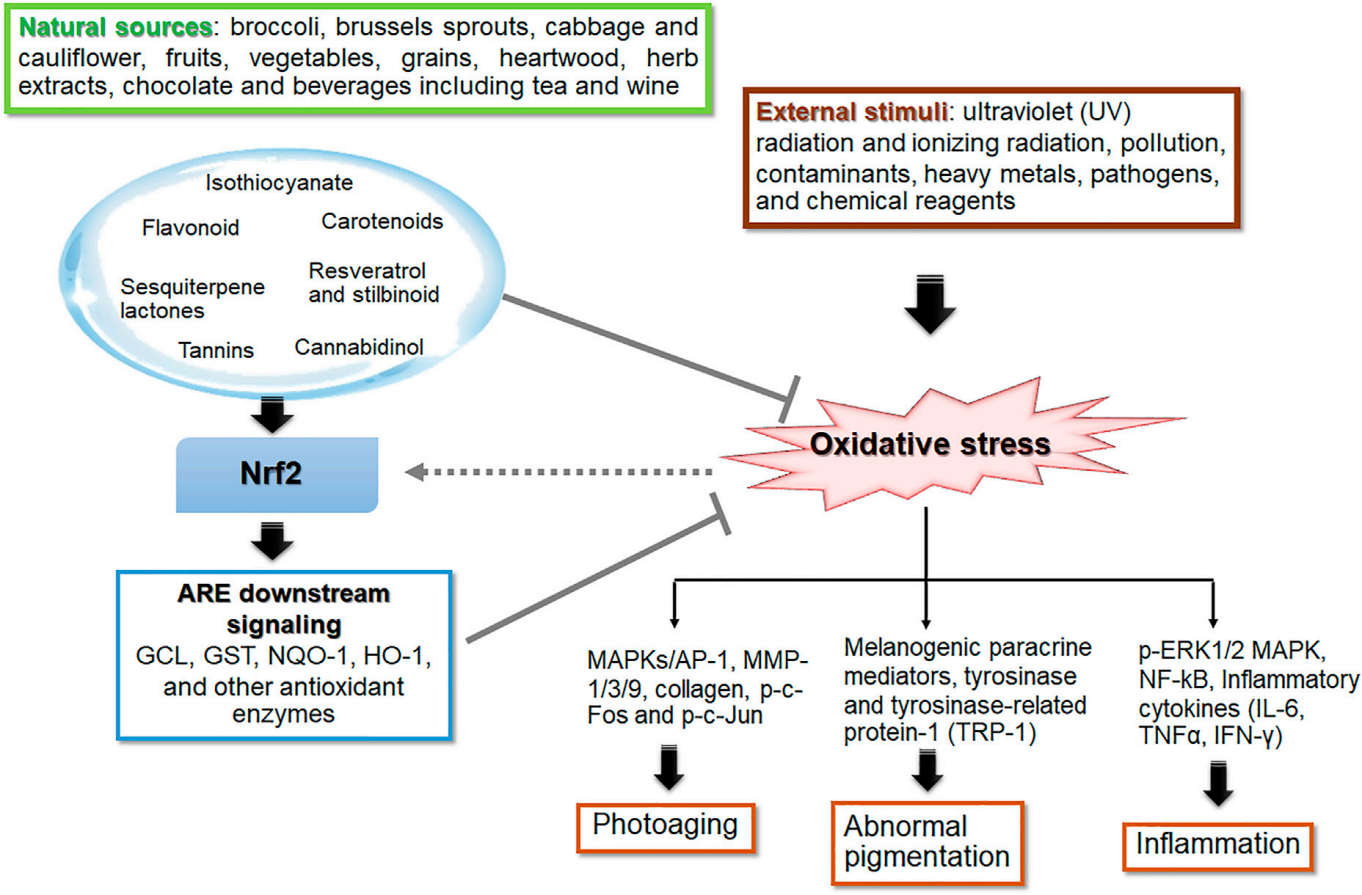
The role of phytochemicals in skin photoprotection *via* regulation of Nrf2 signaling. Phytochemicals are naturally occurring compounds in botanical products which exert biological activities providing medical and nutritional benefit. Oxidative stress, an imbalance in the redox state of the cell, is the result of cellular response to various stimuli including UV and ionizing radiation, pollution, contaminants, heavy metal, pathogens, and chemical reagents. Most of the natural antioxidants are derived from plant materials such as fruits, vegetables, grains, legumes, botanical drugs, spices and plant-based beverages (including tea, coffee, wine and cocoa). The bioactive phytochemicals, e.g., isothiocyanates (in broccoli, brussels sprouts, cabbage and cauliflower), flavonoids, carotenoids, resveratrol and stilbinoid, exhibit a wide range of photoprotective effects, including anti-photoaging, anti-inflammation, and anti-melanogenesis. These protective effects involve activation of nuclear factor erythroid 2-related factor 2 related to the antioxidant response element (Nrf2-ARE) signaling pathway that regulates expression of its downstream target genes including glutamate cysteine ligase (GCL), glutathione S-transferase (GST), NAD(P)H quinone oxidoreductase-1 (NQO-1), heme oxygenase-1 (HO-1) and other antioxidant genes, to cope with various stressors including UVR. The thick arrows and bar-headed lines mean activation and inhibition of the pathway, respectively. The dash arrow means modulation of Nrf2 signaling by ROS levels.

Phytochemicals play a crucial role in photoprotection against UVR-induced skin photodamage *via* UV-absorbing, antioxidant, melanin-modulating, anti-inflammatory properties Antioxidant phytochemicals have been demonstrated to mitigate skin photodamage *in vitro* and *in vivo via* directly scavenging ROS, promoting antioxidant defense capacity, modulating various signaling pathways involved in inflammation, controlling DNA repair, cellular viability and function of the skin (Boo, 2020a; Garg et al., 2020). This review focuses the photoprotective role of phytochemicals in UVR-mediated photoaging, hyperpigmentation and inflammation affecting skin barrier integrity *via* Nrf2-dependent pathway (Figure 2). The phytochemicals as electrophiles can promote cytoprotective proteins and antioxidant defenses *via* upregulating Nrf2 signaling. Keap1 and Cul3 comprise a unique ubiquitin E3 ligase responsible for degradation of Nrf2. Keap1 is a homodimeric protein belonging to the BTB (Broad complex, Tramtrack, Bric-á-brac)-Kelch family of proteins, which are named Kelch-like 1 to 42 (KLHL1–42). The BTB domain of Keap1 is necessary for Keap1 homodimerization and for mediating interactions with cul3/Rbx1 E3 ubiquitin ligase system. The BTB domain contains reactive cysteine residue responsible for interaction with electrophiles and thus plays a crucial role in sensing environmental electrophiles. Post-translational modifications of the highly reactive Cys 151 in Keap1 result in dimerization of Keap1, resulting in loss of Nrf2 ubiquitination, which stabilize the Nrf2 proteasomal degradation (Shin et al., 2020) and subsequent accumulation of Nrf2 and activation of the Nrf2-driven cytoprotective gene machinery (Cleasby et al., 2014). Phytochemicals, which are thiol-reactive electrophiles, covalently bind to the cysteine residue(s) in the dimerization domain of Keap1. Then, the activated ligase complex fails to degrade Nrf2, allowing the transcriptional activation of Nrf2 target genes (Yamamoto et al., 2008). Well-known Nrf2 activators including the isothiocyanate SFN, the alkylating agent iodoacetamide, tBHQ and diethylmaleate were demonstrated to modify C151 in Keap1, that mediates proteasomal degradation, leading to Nrf2 stabilization and enhancing its nuclear accumulation (Deshmukh et al., 2017; Dayalan Naidu et al., 2018; Dayalan Naidu and Dinkova-Kostova, 2020; Taguchi and Yamamoto, 2020).

In addition to the role of phytochemicals as indirect antioxidants by upregulating antioxidant genes, phytochemicals can act as direct antioxidants by their ROS scavenging activities (Dinkova-Kostova and Talalay, 2008). Direct antioxidants, such as (–)-epicatechin-3-gallate and carotenoids (i.e., β-carotene and lycopene) can protect skin cells from ROS-induced damage to the skin cells. Their protective effects are short-lived and involve their abilities to neutralize ROS. ROS is involved in regulating the activity of Nrf2 *via* several mechanisms. Keap1 is considered as a cysteine-based sensor for a variety of endogenous and exogenous stressors including electrophiles and oxidants. ROS is involved in modulate Nrf2 activity *via* Keap-1-dependent and independent mechanisms. For Keap1-dependent mechanism, ROS (e.g., H_2_O_2_) has been demonstrated to promote Nrf2 activity via oxidative modification of Keap1 cysteines, leading to the release of Nrf2 and allowing its nuclear translocation (Espinosa-Diez et al., 2015; Suzuki et al., 2019). For Keap1-independent regulation of Nrf2 activity, MAPKs and glycogen synthase kinase-3 (GSK-3) are suggested to play a role in posttranslational modifications of Nrf2 via phosphorylation accountable for the alterations in its binding to the proteins involved in controlling Nrf2 stability and subsequent transcriptional activity (He et al., 2020). For instance, GSK-3 activation can stimulate Nrf2 nuclear export as well as ubiquitination and degradation, leading to downregulation of the Nrf2/ARE signaling pathway of brain ischemia and reperfusion injury (Chen et al., 2016). In addition, suppression of GSK-3 by activation of upstream phosphoinositide 3-kinase-protein kinase B/Akt (PI3K-PKB/Akt) results in Nrf2 stabilization. Activation of ERK leads to Nrf2 downregulation in diabetic hearts in response to oxidative stress (Tan et al., 2011). Thus, it is possible that ROS which can act as second messengers in protein kinase cascades also have a regulatory role in Nrf2 activity *via* Keap1-independent manner. Phytochemical polyphenols that act as both pro-oxidants through autoxidation to generate ROS (Babich et al., 2011) and direct antioxidants that can increase and decrease cellar ROS levels can affect Nrf2 activity *via* both Keap1-dependent and -independent manners.

**FIGURE 3:**
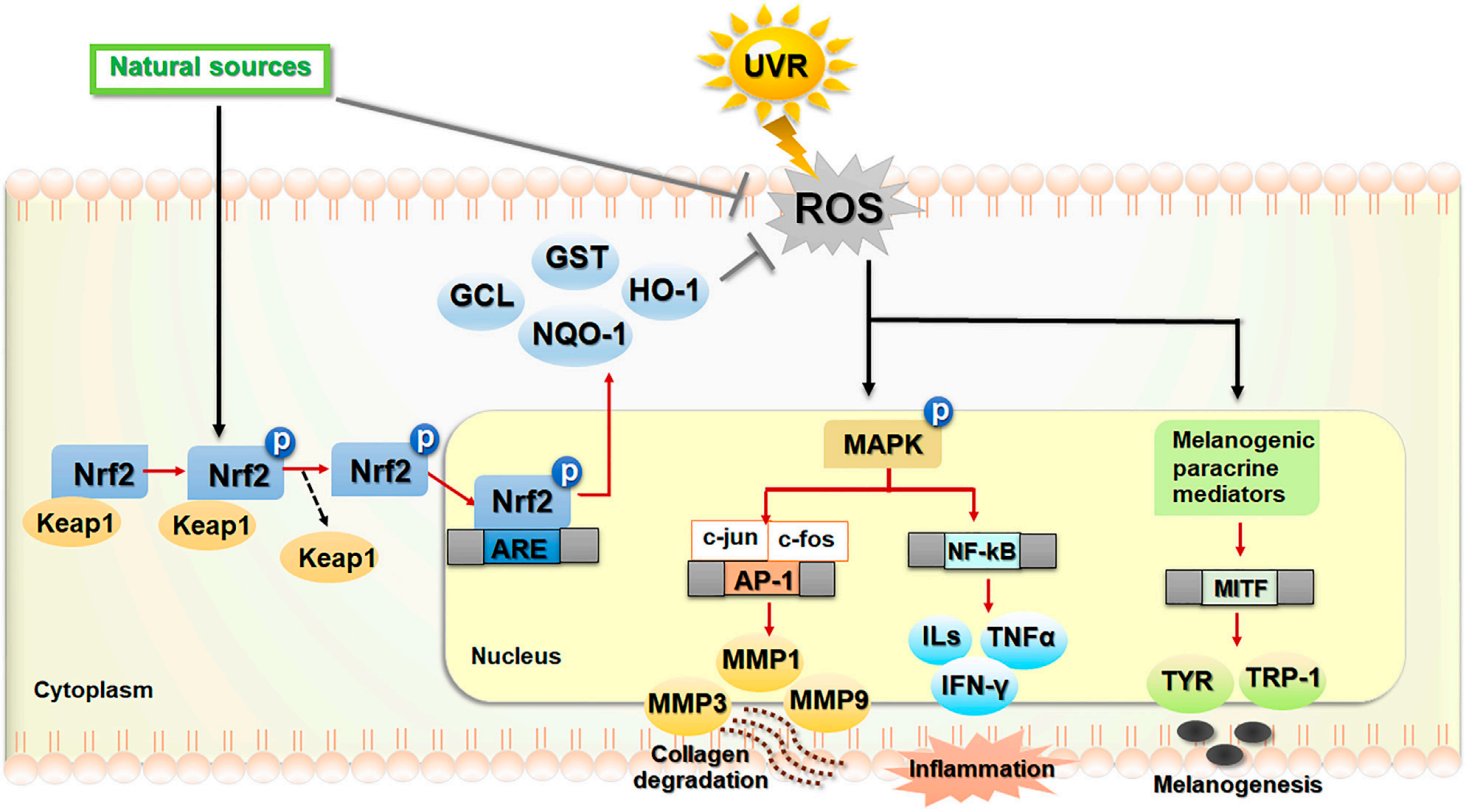
The regulatory mechanism of Nrf2-dependent antioxidant and cytoprotective actions against photoaging, inflammation and hyperpigmentation. Phytochemicals play a photoprotective role against skin photodamage directly *via* scavenging reactive oxygen species (ROS) and indirectly via activation of nuclear factor erythroid 2-related factor 2 (Nrf2) signaling, leading to upregulation of antioxidant and cytoprotective genes. In response to ultraviolet ray (UVR)-induced oxidative stress, ROS, produced in keratinocytes, melanocytes and fibroblasts, can modulate several related signaling pathways involved in photoaging, inflammation and melanogenesis. Exposure of human skin to UVR causes cells to produce ROS, which can modulate the signaling pathways involved in upregulation of matrix metalloproteinases (MMPs) which includes MMP-1/3/9, leading to collagen degradation, inflammation (nuclear factor kappa B also known as NF-κB and its downstream signaling) and the upregulation of melanogenesis-related genes including the microphthalmia-associated transcription factor (MITF), tyrosinase (TYR), and tyrosinase related proteins (TRP-1). Dietary phytochemicals as natural sources of antioxidants play the protective roles against UVR-induced ROS by the inhibition of ROS formation and the activation of Nrf2 signaling. In response to oxidative insults, Nrf2 is activated by the phosphorylation and disassociation of Nrf2 from Kelch-ECH associated protein 1 (Keap1), a repressor protein in the cytoplasm. Then, Nrf2 translocated to the nucleus binds to the ARE in the promoter region of downstream genes encoding antioxidant and phase II detoxifying enzymes including glutamate cysteine ligase (GCL), glutathione S-transferase (GST), NAD(P)H quinone oxidoreductase-1 (NQO-1), heme oxygenase-1 (HO-1). The activation of Nrf2 improves oxidative status of the cells and promotes cytoprotection against skin oxidative damage and inflammation. The black/red arrows and bar-headed lines mean activation and inhibition of the pathway, respectively. The dash arrow means dissociation of the Keap1–Nrf2 complex.

In fact, skin is the largest body organ that is continuously exposed to environmental stressors including UVR. Oxidative stress induced by UVR plays a role in the stress responses of keratinocytes, melanocytes and fibroblasts responsible for photodamaged skin. Hence, development of phytochemicals that can activate the Nrf2 transcription factor is considered a promising pharmacological strategy to prevent and treat UVR-induced skin damage. The phytochemical derivatives that are effective for these photoprotective strategies include polyphenols, flavonoids, non-flavonoids and non-phenolic derivatives. The phytochemicals having ROS scavenging properties could suppress UVB-induced MMP-1 expression in HaCaT cells and human dermal fibroblasts and promote type I procollagen production in human dermal fibroblasts via downregulation of MAPK/AP-1 signaling cascades in association with upregulation of Nrf2 signaling (Kim et al., 2015). Moreover, botanical extracts including extracts of sunflower (*Helianthus annuus* L.) (Hwang et al., 2019), cherry blossoms (Li et al., 2018) and Foeniculum vulgare Mill. (Sun et al., 2016), which are rich sources of antioxidant phytochemicals, were observed to exert the protective effect against UVB-induced ROS formation, MMPs (MMP-1 and MMP-3) production and procollagen type I depletion *via* downregulation of MAPK signaling in association with upregulation of Nrf2 activity in human dermal fibroblasts. The anti-photoaging actions of the sunflower extract were also related to suppression of UVB-induced inflammatory cytokines including IL-6, COX-2, iNOS (inducible nitric oxide synthase), and TNF-α production (Hwang et al., 2019). In addition, the phytochemicals acting as direct or indirect Nrf2 inducers were demonstrated to exert the anti-photoaging effects *via* downregulation of MMPs including MMP-1 *via* MAPK/AP-1 signaling pathways in mouse skin (Sun et al., 2016; Li et al., 2018). Thus, indirect or direct targeting of Nrf2-dependent antioxidant response could offer a promising pharmacological strategy for prevention and inhibition of skin photodamage. The crosstalk between Nrf2 and other signaling pathways (e.g., MAPK/AP-1 pathway) involved in the mechanisms underlying the protective effects of phytochemicals on photodamaged skin were shown in Figure 3 (Kim et al., 2015; Sun et al., 2016; Li et al., 2018; Hwang et al., 2019; Garg et al., 2020). Several *in vitro*, *in vivo* and clinical studies showing the protective roles of botanicals and phytochemicals against photoaging, inflammation, skin barrier dysfunction and hyperpigmentation *via* Nrf2-dependent mechanisms are described below and summarized in Table 1.

**TABLE 1:** The protective roles of botanicals and phytochemicals against photoaging, inflammation, skin barrier dysfunction and hyperpigmentation *via* Nrf2 regulation.

Phytochemicals	Active compounds and sources	Effects	Treatment and study model	Mechanism of action	References
Isothiocyanates	Sulforaphane (SFN), broccoli, brussels sprouts, cabbage and cauliflower	Anti-photoaging	Topical administration of SFN (0.6 μM/cm^2^) for 2 weeks	↓: MMP-1, 8-OHdG DNA damage, MAPKs signaling, c-Jun, and c-Fos	Chaiprasongsuk et al. (2017)
			*In vivo*: BALB/c mice	↑: Nrf2 translocation, Nrf2-target genes, collagen	
	SFN, broccoli, brussels sprouts, wasabi	Anti-inflammation and improvement of skin barrier function	Topical administration of SFN (2.5–10 mg/kg)	↓: Janus kinase 1/STAT3 signaling, skin thickness and eosinophil accumulation in atopic dermatitis mouse skin lesions	Wu et al. (2019)
			*In vivo*: BALB/c mice	↑: Nrf2 and Nrf2-dependent antioxidant enzymes (HO-1)	
	SFN	Hyper-pigmentation	B16F10 cells treated with 10 µM of SFN for 6 h	↓: Tyrosinase activity, melanin content, ROS and 8-OHdG	Chaiprasongsuk et al. (2016)
				↑: Nrf2 and Nrf2-dependent antioxidant enzymes (GCL, GST, NQO-1)	
Flavonoids	Flavanones (Hesperetin; HSP)	Anti-photoaging	Topical administration of HSP (0.3, 1, and 3 mg/cm^2^) for 2 weeks	↓: MMP-1, 8-OHdG DNA damage, MAPKs signaling, c-Jun, and c-Fos	Lohakul et al. (2021a)
			*In vivo*: BALB/c mice	↑: Nrf2 translocation, Nrf2-target genes, collagen	
	Fisetin	Anti-inflammation and improvement of skin barrier function	Topical administration of Fisetin (25–100 µM) for 10 weeks	↓: pro-inflammatory mediators (COX-2, IL-6, and NF-κB), aquaporin and filaggrin (the protein markers of skin barrier function)	Wu et al. (2017)
			*In vivo*: BALB/c mice	↑: Nrf2	
	Caffeic acid, ferulic acid, quercetin and rutin	Hyper-pigmentation	Treatment of cells with of caffeic acid, ferulic acid, quercetin and rutin prior to UVA irradiation	↓: Melanogenesis	Chaiprasongsuk et al. (2016)
			*In vitro*: B16F10 melanoma cells	↑: Nrf2 and Nrf2 targeted genes	
	Ellagic acid		Treatment with ellagic acid (20–80 μM) for 24–72 h	↓: protein levels of the paracrine factors, proopiomelanocortin (POMC), α-MSH, and AKT/JNK/ERK signaling	Yang et al. (2021)
			*In vitro*: keratinocyte HaCaT cells	↑: Nrf2 nuclear protein	
Carotenoids	Rosemary extract (carnosic acid)	Anti-photoaging	Pre-treatment with rosemary extract containing carnosic acid (2.5–10 µM) for 6–9 h	↓: matrix metalloproteinases (MMPs)	Calniquer et al. (2021)
			*In vitro*: HaCaT keratinocytes and KERTr keratinocytes	↑: Nrf2/ARE systems	
	Crystalline lycopene preparations purified from tomato extract (>97%), carotenoid-rich Tomato Nutrient Complex (TNC), rosemary extract	Anti-inflammation and improvement of skin barrier function	Pre-treatment with the different compounds at a concentration of 5 µM	↓: NF-κB activity and IL-6	Calniquer et al. (2021)
			*In vitro*: HaCaT keratinocytes	↑: ARE/Nrf2 activity	
	Fucoxanthin		topical application of cream containing the fucoxanthin (0.2% w/w) to mouse skin	↓: melanin index and skin edema, COX and IL-6	Rodriguez-Luna et al. (2018)
			*In vivo*: Female Swiss CD-1 mice	↑: Nrf2-dependent antioxidant enzymes; heme oxygenase-1 (HO-1)	
Resveratrol and stilbenoid	Grape peel extract, dried heartwood of *Pterocarpus marsupium* Roxb.	Anti-photoaging	Oral administration of either 2 g GPE or 2 mg resveratrol per kg body weight in mice	↓: skin wrinkle formation	Kim et al. (2019)
			*In vivo*: mice	↑: Nrf2-dependent antioxidant enzymes; heme oxygenase-1 (HO-1)	
	Resveratrol	Hyper-pigmentation	Treatment of UVB-irradiated skin with resveratrol	↓: MITF and its target proteins including TYR, TRP1, TRP2	Lee et al. (2014)
			*In vivo*: Guinea Pig Skin	↑: Nrf2/HO-1 proteins	
	Pterostilbene extracted from the dried heartwood of *Pterocarpus marsupium* Roxb.		Treatment with pterostilbene	↓: melanogenesis and tyrosinase activity	Li et al. (2017a), Majeed et al. (2020)
			*In vitro*: B16F10 mouse melanoma cells	↑: Nrf2-mediated HO-1, γ-GCLC, and NQO-1 protein expressions	
			Keratinocyte HaCaT cells		
	Pterostilbene (Pter)	Anti-inflammation and improvement of skin barrier function	Pre-treatment with Pter (5 and 10 μM) for 24 h prior to UVB irradiation (300 mJ/cm^2^)	↓: ROS generation	Li et al. (2017a)
			*In vitro*: HaCaT keratinocytes	↑: Nuclear translocation and phosphorylation of Nrf2, expression of Nrf2-dependent antioxidant enzymes, DNA repair activity, phosphatidylinositol-3-kinase (PI3K) phosphorylated kinase, Akt	
Sesquiterpene lactones	Santamarine isolated from Asteraceae scoparia, artichoke (*Cynara scolymus* L.)	Anti-photoaging	Pre-treatment with Santamarine (1–10 µM) for 1 h	↓: ROS levels, MAPKs/AP-1, and MMP-1/3/9, p-c-Fos and p-c-Jun	Oh et al. (2021)
			*In vitro*: human dermal fibroblasts	↑: collagen I, TGF-β/Smad signaling, Nrf2-dependent intracellular antioxidant mechanism (SOD and HO-1)	
	Cynaropicrin (Cyn)	Anti-inflammation and improvement of skin barrier function	Pre-treatment with Cyn (up to 100 μM) prior to UVB irradiation (50 mJ/cm^2^)	↓: ROS generation, TNF-α, BaP	Takei et al. (2015)
			*In vitro*: Normal human epidermal keratinocyte (NHEKs)	↑: Nrf2, Nrf2-dependent antioxidant enzymes (NQO-1)	
Tannins	Red raspberries (*Rubus idaeus* L.) extracts	Anti-photoaging	Pre-treatment with *Rubus idaeus* L. (1–100 *µ*M) for 1 h	↓: MAPK/AP-1, NF-κβ and TGF-β/Smad	Gao et al. (2018b)
			*In vitro*: normal human dermal fibroblasts (NHDFs)	↑: type I procollagen and Nrf2 nuclear transfer	
	*Alchemilla mollis* (Buser) Rothm. (AM) extract		Treatment with AM (1, 10 and 100 μg/ml) for 4 h after UVB irradiation (144 mJ/cm^2^)	↓: ROS production, TGF-β1, MMP-1, IL-6, and nucleus NFATc1 dephosphorylation, wrinkle formation, skin thickening, water loss, and erythema	Hwang et al. (2018)
			*In vitro*: NHDFs	↑: type I procollagen and elastin expression, Nrf2-dependent antioxidant enzymes (NQO-1 and HO-1)	
			*In vivo*: hairless mice		
	Red raspberries extract (RBE)	Anti-inflammation and improvement of skin barrier function	Pre-treatment with RBE (62.5–1,000 μg/ml) for 48 h prior to UVB irradiation (100 mJ/cm^2^)	↓: Cell viability, epidermal thickness	Wang et al. (2019)
			Topical treatment with RBE (750 μg/ml)	↑: Nrf2, Nrf2-dependent antioxidant enzymes (catalase, SOD, NQO-1, and HO-1)	
			*In vitro*: HaCaT cells		
			*In vivo*: nude mice (ICR-Foxn/nu strain)		
Terpenoids: diterpene, triterpene and sesquiterpene	Ginsenosides compound Mx (C-Mx) from Notoginseng stem-leaf	Anti-photoaging	Pre-treatment with the Ginsenosides C-Mx (1–20 uM) for 3–72 h	↓: MMP-1 and 3	Liu et al. (2018)
			*In vitro*: NHDFs were obtained via skin biopsy from a healthy young male donor	↑: Nrf2, Nrf2-dependent antioxidant enzymes (NQO-1 and HO-1), procollagen	
	Ginsenoside Rg1	Anti-inflammation and improvement of skin barrier function	Pre-treatment with Rg1 (50 μM) for 1 h	↓: IL6 and 8	Li et al. (2016)
			*In vitro*: HaCaT cells	↑: Nrf2, Nrf2-dependent antioxidant enzymes (GCLC, GCLM, and HO-1)	
	Ginsenoside C-Y, a Ginsenoside Rb2 Metabolite from American Ginseng	Hyper-pigmentation	Pre-treatment with C-Y (10, 20, 30 lM) for 72 h	↓: melanin content and tyrosinase activity	Liu et al. (2019)
			*In vitro*: NHDFs	↑: Nrf2, Nrf2-dependent antioxidant enzymes (NQO-1 and HO-1)	

### Isothiocyanates

Isothiocynates are sulfur-containing compounds having the general formula R–N=C=S and are commonly found in cruciferous vegetables from the *Brassica* genus including broccoli, brussels sprouts, cabbage and cauliflower. Members of isothiocyanates widely known as Nrf2 activators including SFN and phenethyl isothiocyanate (PEITC) protected human *ex vivo* full skin against UVR-induced sunburn cells, apoptosis and the decreased activity of the antioxidant enzyme catalase in correlation to upregulation of Nrf2 activity and its target genes (γ-GCS, HO-1, NQO-1) in HaCaT keratinocytes (Kleszczynski et al., 2013). SFN (0.6 μM/cm^2^) was observed to exert anti-photoaging effects on mouse skin via inhibition of MAPK/AP-1 signaling in UVA-irradiated mouse skin (Chaiprasongsuk et al., 2017). Treatment of keratinocyte cell line NCTC2544 with SFN (10 µM) combined with the Fernblock® XP (1 and 2 mg/ml), obtained from the tropical fern *Phlebodium aureum* (L.) J.Sm., substantially suppressed the production of MMP-1, MMP-3 and IL-1 in association with a decrease in ROS production. The SFN (5 and 10 µM) and Fernblock® XP (1 mg/ml) combination also showed inhibitory effects on melanoma cell growth and migration *in vitro* in association with the ability to inhibit the inflammatory microenvironment and neo-angiogenesis (Serini et al., 2020).

Benzyl isothiocyanate and 6-(Methylsulfinyl)hexyl isothiocyanate derived from Wasabi, have been reported to suppress a transcriptional levels of COX-2, an enzyme synthesizing the pro-inflammatory mediators (Lee et al., 2009; Uto et al., 2012). The disturbance of phosphorylated MAPKs signaling, ERK, p38 kinase, and JNK, was observed in the ITCs treatment, resulting in the downregulation of the transcription of inflammatory genes such as COX-2, iNOS, TNF-α, IL-1β, and IL-6 (Lee et al., 2009; Latronico et al., 2021). The effects of allyl-isothiocyanate and SFN on the Nrf2 nuclear translocation were associated with the downregulation of p65 protein, a subunit of the transcription factor NF-κB (Wagner et al., 2012). A previous study also suggested a connection between activation of Nrf2 and expression of keratin 16, a key intermediate cytoskeletal protein responsible for maintaining the skin barrier integrity in response to injury or inflammation. While genetic deletion of Nrf2 caused an early onset of hyperkeratotic lesions in *Krt16* null mice which developed palmoplantar keratoma, topical treatment with SFN prevented the development of the skin lesions in footpad skin in association with restoring redox balance (Kerns et al., 2016).

Isothiocyanates including SFN and 7-methylsulfonylheptyl isothiocyanate (7-MSI), the sulfur-rich phytochemicals found in cruciferous vegetables, have been observed to exert anti-melanogenic effects via downregulation of MAPKs, the main regulatory pathways of melanogenesis (Shirasugi et al., 2010). 7-MSI treatment significantly reduced melanogenesis in B16F1 melanoma cells *via* activation of ERK signaling, leading to activation of autophagy and downregulation of MITF, tyrosinase and TRP-1 (Kim et al., 2021). Additionally, SFN exerted protective effects on particulate matter-induced melanogenesis *via* decreasing the release of paracrine factors by keratinocytes (Ko et al., 2020). Our previous study also revealed that the mechanisms underlying the anti-melanogenic effects of SFN involved the activation of Nrf2-mediated antioxidant response (Chaiprasongsuk et al., 2016).

### Flavonoids

Flavonoids are most abundant polyphenols found in fruits, vegetables, grains, chocolate and beverages including tea and wine. This group has a common basic structure consisting of two aromatic rings bound together by three carbon atoms forming an oxygenated heterocycle. The flavonoids include flavanols (e.g., catechin, epicatechin), flavonols (e.g., quercetin, kaempferol, rutin), flavanones (e.g., hesperetin), flavones (e.g., apigenin, luteolin, hispidulin), isoflavones (e.g., daidzein, genistein) and anthocyanins (e.g., cyanidin) (Panche et al., 2016).

Rutin (Q-3-*O*-rutinoside) is a flavonol glycoside abundantly found in plants such as tea, onions, wine, apples and berries. Previous studies reported the protective role of rutin in aging on human dermal fibroblasts (HDFs) *via* upregulation of collagen type 1 and downregulation of *MMP-1* mRNA in HDF. Application of the rutin-containing cream also improved skin elasticity as well as length and area of crow’s feet (Choi et al., 2016). Furthermore, the analysis of proteome profiles revealed that rutin treatment caused an induction of proteins involved in the antioxidant defenses and a reduction of proteins involved in the degradation of Nrf2 in UVB-irradiated dermal fibroblasts (Gegotek et al., 2018). Hesperidin, (hesperetin-7-rutinoside), and its aglycone, hesperetin, mostly found in citrus fruits and botanical drugs, have been demonstrated to provide *in vitro* and *in vivo* anti-photoaging effects on the skin *via* stimulating collagen synthesis in association with the antioxidant properties (Lohakul et al., 2021a). Citrus sinensis peel extract containing hesperidin loaded lipid nanoparticles showed photoprotective effects on UVR-mediated induction of MMP-1 *via* JNK signaling, reduction of collagen accompanied by decreased SOD protein production as well as stimulated inflammatory markers (COX-2, prostaglandin E2) and lipid oxidation product, malondialdehyde (MDA), in mouse skin (Amer et al., 2021). It was also suggested that the protective effect of a mixture of methylated derivatives of hesperidin on UVB-induced skin damage might involve the abilities to promote Nrf2 nuclear translocation and mRNA levels of its target gene GCLC and HO-1 in keratinocytes (Kuwano et al., 2015). In addition, a clinical trial showed that a 12-week topical application of a serum containing 0.1% hesperetin significantly promoted skin hydration and elasticity *via* enhancing the synthesis of hyaluronic acid (Sheen et al., 2021). Moreover, hesperetin and polyherbal formula extracts containing hesperetin topically applied to mouse skin before UVA exposure three times per week for 2 weeks (a total dose of 60 J/cm^2^) significantly attenuated MMP-1 upregulation and collagen depletion concomitant with promoting Nrf2 activity and the level of its target proteins (GST and NQO-1) as well as reducing 8-hydroxy-2′-deoxyguanosine (8-OHdG), a product of oxidatively damaged DNA damage, in irradiated mouse skin (Lohakul et al., 2021a). Grape fruit stem extract from Muscat Bailey A containing catechin, epicatechin and trans-resveratrol showed protective effects on UVB-induced destruction of collagen fiber through reduction of MMP-1 expression in association with a decrease in lipid peroxidation and restoration of GSH levels in mouse skin (Cho et al., 2018).

Furthermore, flavonoids consequently affect immune mechanisms that are essential in the development of the inflammatory processes (Maleki et al., 2019). Treatment of human epidermal keratinocytes (HaCaT cells) with 6-shogaol, an active ingredient of ginger, resulted in suppressing the UVB (180 mJ/cm^2^)-induced iNOS, COX-2 and TNF-α, which are the key mediators of inflammatory response, through modulating Nrf2 signaling (Wu et al., 2011; Du et al., 2018; Chen et al., 2019). In addition, the flavonoids have been suggested to exert anti-inflammatory actions in association with Nrf2 activation *in vitro* and *in vivo*. The mechanisms underlying the anti-inflammatory effects of flavonoids involved inhibition of production of pro-inflammatory cytokines including IL-33, TNF-α, IL-1β, IL-6 and downregulation of NF-κB activity (Staurengo-Ferrari et al., 2018). Gallocatechin-silver nanoparticle was observed to improve wound healing in diabetic rats via inhibiting TLR4/NF-κB inflammatory signaling pathway and modulating Nrf2/HO-1 pathway (Ni et al., 2015). The flavonol Galangin, obtained from *Alpinia officinarum* Hance and propolis extracts, was able to mitigate imiquimod-induced psoriasis-like skin inflammation in BALB/c mice *via* inhibiting pro-inflammatory mediators of COX-2, iNOS, NF-κB pathway and pro-inflammatory cytokines IL-17, IL-23, IL-1β in the skin as well as IL-6, TNF-α in both skin and serum. The anti-inflammatory effects of galangin were also associated with its ability to induce Nrf2 activity (Sangaraju et al., 2021). Recent evidence has revealed that the flavone luteolin improved impaired healing and promoted re-epithelization of skin wound in streptozotocin-induced diabetic rats *via* suppressing expressions of inflammatory proteins including MMP-9, TNF-α, IL-6, IL-1β and downregulating NF-κB in association with activation of Nrf2-dependent upregulation of antioxidant enzymes (Chen LY. et al., 2021). Topical application of a flavonoid fisetin (50 and 200 µM) for 10 weeks to mouse skin after UVB exposure was demonstrated to mitigate skin photodamage by inhibiting MMP-1 and MMP-2 protein expressions and collagen degradation as well as by improving skin barrier dysfunction. The fisetin treatment resulted in restoring skin hydration and barrier function in UVB-irradiated mouse skin through promoting contents of filaggrin, a structural protein in the stratum corneum, and aquaporins, integral epidermal cell membrane proteins, responsible for epidermal hydration and barrier function. The anti-photodamaging effects of fisetin are suggested to involve upregulation of Nrf2 and downregulation of pro-inflammatory mediators (COX-2, IL-6, and NF-κB) (Wu et al., 2017).

We previously observed that caffeic acid, ferulic acid, quercetin and rutin provided anti-melanogenic effects *via* enhancing Nrf2-mediated antioxidant defense responses in UVA-irradiated B16F10 cells (Chaiprasongsuk et al., 2016). Ellagic acid was shown to suppress α-MSH-induced melanin synthesis and tyrosinase activity by downregulating cAMP-mediated CREB and MITF signaling in B16F10 cells. Ellagic acid also had ability to suppress protein levels of the paracrine factors, POMC and α-MSH, through Nrf2 activation in keratinocyte HaCaT cells (Yang et al., 2021).

Licorice root extracts have traditionally been used for skin problems and are suggested as one the top cosmeceutical ingredients for hyperpigmentation (Searle et al., 2020). The root and rhizome extracts of licorice and several flavonoids identified as its bioactive ingredients have been suggested to provide beneficial effects on the skin through tyrosinase inhibitory activity, ROS scavenging activity, anti-inflammatory activity and Nrf2 inducing activity (Ciganovic et al., 2019). Glycyrrhiza flavonoids and licochalcone A, a major component of the licorice root extracts, showed the inhibitory effects on melanogenesis via activation of ERK and the subsequent downregulation of MITF/tyrosinase pathway in B16F10 cells. Isoliquiritigenin, a flavonoid component from the hydrolysis products of licorice root, was observed to exert anti-melanogenic effects on α-MSH-, ACTH- and UV-induced melanin synthesis and on melanocyte dendricity and melanosome transport through downregulation of melanogenic proteins including tyrosinase, TRP-1, DCT, Rab27a and Cdc42 in melanocytes (Lv et al., 2020).

### Tannins

There are three major classes of tannins: condensed tannins (e.g., proanthocyanidins, flavonol-based compounds); hydrolyzable tannins (gallotannins and ellagitannins) and phlorotannins. Gallic acid, a chemical constituent of tannins, and its derivatives are found in almost all organ of a plant including bark, wood, leaf (in particular tea leaves), fruit, root and seed.

Red raspberries (*Rubus idaeus* L.) extracts containing high levels of anthocyanins and ellagitannins including Sanguiin H-6 and lambertianin C showed the protective effect on UVB-induced photoaging in normal human dermal fibroblasts (NHDFs). Treatment with the red raspberry extracts resulted in a significant reduction of MMPs secretion and production of pro-inflammatory mediator IL-6 possibly via downregulating MAPK, NF-κβ and AP-1 as well as increased procollagen type I production *via* activating the TGF-β/Smad pathway. The anti-photoaging effects of the tannin-rich botanical drugs involved promotion of Nrf2 activity and its target antioxidants including HO-1 and NQO-1 (Gao et al., 2018b). *Alchemilla mollis* (Buser) Rothm. ethanolic extract possessing gallic acid showed protective effects on UVB-induced photoaging in NHDFs and in mouse skin *in vivo*. Treatment with *Alchemilla mollis* (Buser) Rothm. ethanolic extract led to a substantial reduction in ROS formation as well as MMP-1 and IL-6 promotion through downregulating AP-1 activity in NHDFs exposed to UVB (144 mJ/cm^2^) irradiation. Furthermore, treatment with *Alchemilla mollis* (Buser) Rothm. extract and gallic acid protected against UVB-induced a reduction of type I procollagen levels in association with promotion of TGF-β1 *in vitro* and *in vivo*. Oral administration of *Alchemilla mollis* (Buser) Rothm. extract and gallic acid also improved UVB-induced wrinkle formation, skin dryness, epidermal thickening and collagen fiber density in hairless mice. The antioxidant mechanism underlying the anti-photoaging effects of *Alchemilla mollis* (Buser) Rothm. extract also involved the upregulation of Nrf2/HO-1 pathway (Hwang et al., 2018). Black tea (Fuzhuan-brick tea, rich in gallic acid and tea polyphenols) and gallic acid were also demonstrated to provide anti-photoaging effects *via* upregulation of Nrf2/HO-1 signaling in association with activation of MAPK signaling (p38 and ERK1/2 phosphorylation) in UVB-exposed keratinocyte HaCaT cells (Zhao et al., 2018). Cocoa phytochemicals including procyanidins and the flavanol catechin and epicatechin have been suggested to have several biological activities including antioxidant and anti-inflammatory activities possibly responsible for their beneficial effects on various age-related diseases including skin aging (Kim et al., 2014). Long term consumption of cocoa beverage for 12 weeks protected against UV-induced skin erythema and improved skin conditions (including erythema, skin roughness and scaling) in women (Heinrich et al., 2006). Procyanidins showed abilities to activate Nrf2 signaling in various *in vitro* and *in vivo* models (Truong et al., 2014). Therefore, it is possible that mechanisms underlying the anti-photoaging effects of procyanidins involve activation of Nrf2-regulated antioxidant defenses.

Phlorotannins (PTNs) are tannins found primarily in brown algae and play a role in protecting cells against UVR. PTNs applied topically attenuated radiation-induced inflammatory responses by downregulating NF-κB signaling and its downstream COX-2 and inflammasome activation in a mouse model of radiation dermatitis. PTNs also showed the abilities to promote wound healing process by enhancing aquaporin 3 involved in epidermal hydration and homeostasis. The mechanisms underlying the anti-inflammatory and wound healing-promoting effects of PTNs on irradiated mouse skin also involved upregulation of Nrf2/HO-1 signaling (Yang et al., 2020).

Gallic acid, gallotannin, valonia tannin and extracts of plants (e.g., Ceylon olive leaves and pomegranate peel) containing gallic acid exerted antimelanogenic effects directly by acting as a competitive inhibitor of tyrosinase and indirectly by inhibiting tyrosinase *via* antioxidant actions that affect melanogenesis pathway *in vitro* and *in vivo* (Chen et al., 2009; Panich et al., 2013; Su et al., 2013; Kanlayavattanakul et al., 2020; Huang et al., 2021; Liu et al., 2021). A randomized, double-blind, placebo-controlled clinical trial demonstrated that continuous administration of apple polyphenol rich in procyanidins for 12 weeks improved UV-induced skin pigmentation in heathy women (Shoji et al., 2020). Our previous study demonstrated that gallic acid protected against UVA (8 J/cm^2^)-induced melanogenesis via modulation of Nrf2 signaling and promotion of antioxidant defenses (including GSH, catalase, GPx and GST) in B16F10 melanoma cells (Onkoksoong et al., 2018).

### Resveratrol and its Derivatives

Resveratrol was observed to provide photoprotective effects in UVB-induced photoaging *via* the antioxidant, anti-inflammatory and antiapoptotic actions in human keratinocyte HaCaT cells and ICR mice *in vivo*. The mechanism underlying its antiphotoaging actions involves upregulation of Nrf2 signaling and the antioxidant defenses (including HO-1, NQO-1, SOD1, GPx-4) in association with suppression of aging markers (MMP-1 and MMP-9) and proinflammatory mediators (IL-6, TNF-*α*, VEGF-B) by inhibiting ROS-mediated MAPK and COX-2 signaling cascades (Cui et al., 2022). Oral administration of grape peel extract and resveratrol exerted the anti-photoaging effects on UVB-induced skin wrinkle formation *via* promotion of Nrf2/HO-1 signaling cascades (Kim et al., 2019). In addition, a formulation containing 0.4% pterostilbene, the resveratrol analog, extracted from the dried heartwood of *Pterocarpus marsupium* Roxb., showed substantial reduction of aging markers and improvement of wrinkles, skin hydration elasticity in healthy volunteers (Majeed et al., 2020).

Topical treatment with pterostilbene, the resveratrol analog, suppressed an acute UVB radiation-induced skin inflammation and prevented chronic UVB radiation-mediated carcinogenesis in mice. The mechanisms involved in the photoprotective effects of pterostilbene might be attributed its ability to absorb UVB, protect against oxidative damage to DNA, protein and lipid, promote activities of antioxidant enzymes including catalase, SOD and GPx as well as activate Nrf2-dependent antioxidant response (Pastore et al., 2012; Sirerol et al., 2015). Moreover, resveratrol was observed to promote wound healing by restoring cell proliferation and migration, along with increased Nrf2 activity and Mn-SOD expression in the diabetic rat model (Zhou et al., 2021).

Resveratrol acting as a direct antioxidant, Nrf2 activator and tyrosinase inhibitor has been suggested to suppress melanogenesis (Wang et al., 2018; Boo, 2019). Resveratrol treatment led to a substantial reduction of UVB-induced melanogenesis *via* downregulation of MITF and its target proteins including TYR, TRP1, TRP2 in correspondence to upregulation of Nrf2/HO-1 proteins in melanocytes (Jian et al., 2011). Topical administration of 0.4% resveratryl triacetate and 0.4% resveratryl triglycolate twice daily for up to 8 weeks after the artificial pigmentation was shown to provide whitening efficacy in human subjects (Ryu et al., 2015; Jo et al., 2018). Pterostilbene, a stilbinoid, found in blueberries and grapes was demonstrated to exert anti-melanogenic effects *via* promotion of autophagy in melanocytes and downregulation of CREB (cAMP response element-binding protein)-MITF-tyrosinase pathway in B16F10 cells treated with HaCaT conditioned medium. The protective effects of pterostilbene on melanogenesis involved suppression of UVA-induced α-MSH expression and upregulation of Nrf2-mediated HO-1, γ-GCLC, and NQO-1 protein expressions in HaCaT cells (Hseu et al., 2021). Additionally, the application of a 0.4% formulation of natural pterostilbene for 4 and 8 weeks showed the skin brightening and anti-aging effects, respectively, in healthy volunteers in an open-label, single-arm, monocentric efficacy study (Majeed et al., 2020).

### Carotenoids

Carotenoids, which belong to the tetraterpenes family, are liposoluble pigments responsible for the yellow, orange or red color of fruits, leaves and flowers. The carotenoids are divided into carotenes, xanthophylls and lycopene, and are abundantly present in tomato, carrots, pumpkin, seaweeds and algae (Milani et al., 2017). The main carotenoids including astaxanthin, canthaxanthin, *β*-carotene, lycopene and lutein have been suggested to exert photoprotective effects against UVR-induced skin photodamage *via* inhibition of inflammatory responses and photoaging biomarkers as well as promotion of antioxidant defense system in several *in vitro*, *in vivo* and clinical studies (Aust et al., 2005; Camera et al., 2009; Cavinato et al., 2017; Cooperstone et al., 2017; Kohandel et al., 2022). Previous reports have suggested that the carotenoids including astaxanthin and lycopene exert pharmacological activities including antioxidant, anti-inflammatory and chemopreventive activities against skin damage *via* activation of Nrf2 signaling (Wang et al., 2020; Ahn and Kim, 2021; Kohandel et al., 2021).

Dietary carotenoids combined with rosemary extract containing carnosic acid having abilities to activate Nrf2/ARE system exerted an inhibitory effect on UVB (20–60 mJ/cm^2^)-induced TNF-α and MMP-1 secretion from dermal fibroblasts (Calniquer et al., 2021). The tomato extracts rich in lycopene also protected against H_2_O_2_-induced photodamage of fibroblasts via promotion of pro-collagen secretion and suppression of apoptotic cell death and ROS formation (Darawsha et al., 2021).

Lutein, a xanthophyll carotenoid obtained from green leafy vegetables and egg yolk, has been reported to exert the anti-inflammatory effects *via* modulation of oxidant-sensitive inflammatory signaling pathways including NF-κB and STAT3 pathways and suppression of inflammatory cytokines (such as IL-1β, IL-6, TNF-α, COX-2, iNOS) (Ahn and Kim, 2021). Astaxanthin was demonstrated to exert anti-inflammatory effects by suppressing the expression of pro-inflammatory cytokines, for example, COX-2, LOX-1, NF-κB p65, TNF, and IL-1 (Kishimoto et al., 2010). Additionally, combinations of tomato nutrient complex containing lycopene and carotenoids and rosemary extract containing carnosic acid were observed to protect against UVB-induced oxidative stress, photoaging and inflammatory responses by inhibition of NF-κB activity and IL-6 production, along with activation of the ARE/Nrf2 system using HaCaT and KERTr keratinocyte cell lines (Calniquer et al., 2021). Bixin, an apocarotenoid isolated from the Achiote (*Bixa Orellana* L.), is one of the most consumed food colorants and topical preparations of seed extracts of the achiote have been in ethno-pharmacological use for treatment of wound healing and the pathologies related to epithelial barrier disruption. The mechanisms underlying the pharmacological actions of bioxin in improving skin barrier function were suggested to involve activation of Nrf2-mediated antioxidant systems including thioredoxin (TRX)/thioredoxin reductase (TXNRD1), regulation of peroxisome proliferator-activated receptors (PPARs), responsible for skin homeostasis and epithelial repair and modulation of Toll-like receptor 4 (TLR4)/NF-κB inflammatory signaling pathway (Rojo de la Vega et al., 2017). The marine carotenoid fucoxanthin (found in brown seaweeds, the microalgae and diatoms) showed anti-inflammatory actions through inhibiting proinflammatory cytokines including TNF-α, IL-6 and IL-8 levels as well as suppressing UVB-mediated oxidative stress in keratinocyte HaCaT cells. Moreover, topical application of cream containing the fucoxanthin to mouse skin protected against UVB-induced skin inflammation and hyperplasia *via* downregulation of COX-2 and iNOS, along with upregulation of Nrf2 activity and its target protein HO-1 (Rodriguez-Luna et al., 2018).

### Terpenoids: Diterpene, Triterpene and Sesquiterpene

Santamarine, a sesquiterpene lactone, isolated from sunflower family provided anti-photoaging effects via suppression of UVA (8 J/cm^2^)-induced MAPK/AP-1 pathways involved in upregulation of MMPs and *via* promotion of TGF-β/Smad-mediated collagen production in HDFs. Furthermore, santamarine treatment led to a significant restoration of UVA-mediated downregulation of Nrf2-dependent antioxidant defenses including SOD-1 and HO-1 at the mRNA and protein levels (Oh et al., 2021). Zerumbone (ZER), a natural sesquiterpene, from *Zingiber zerumbet* (L.) Roscoe ex Sm. rhizomes was demonstrated to protect against UVA irradiation (3 J/cm^2^)-induced ROS formation, MMP-1 activity and collagen III degradation in HDFs. ZER was suggested to exert the anti-photoaging effects *via* downregulation of AP-1 activity and promotion of Nrf2/ARE pathway (Hseu et al., 2019). Furthermore, treatment of dermal fibroblasts with rosemary extracts rich in the diterpene carnosic acid having abilities to induce ARE/Nrf2 reporter activity protected against TNF-α-induced MMP-1 secretion (Calniquer et al., 2021). Ginsenosides, triterpene saponins, are major bioactive compounds responsible for pharmacological activities of Panax (ginseng), which has traditionally been used to treat and prevent various conditions associated with aging including skin aging. Previous studies demonstrated that the rare minor ginsenosides (C-Mc and Mx), which may act as potential antiphotoaging compounds, suppressed MMP production *via* regulating MAPK/AP-1/NF-κB pathway and promoted collagen production via the TGF-β/Smad pathway in association with upregulation of Nrf2 signaling in UVB-irradiated human dermal fibroblasts (Liu et al., 2018; Liu et al., 2022).

Cynaropicrin, a sesquiterpene lactone, is the major bioactive phytochemical in the artichoke (*Cynara cardunculus* L.) that can activate aryl hydrocarbon receptor (AhR), resulting in nuclear translocation of Nrf2. Activation of AhR/Nrf2/NQO-1 pathway by cynaropicrin was involved in its inhibitory effects on UVB-mediated production of proinflammatory cytokines including IL-1 and TNF-α in keratinocytes (Takei et al., 2015). Hemistepsin A, a sesquiterpene lactone isolated from *Saussurea lyrata* (Bunge) Franch., has been demonstrated to exert pharmacological actions including anti-inflammatory and antioxidant activities. Treatment of keratinocyte HaCaT cells with hemistepsin A protected against H_2_O_2_-induced cytotoxicity, DNA damage and apoptosis-mediated by mitochondrial dysfunction via upregulation of Nrf2/HO-1 signaling pathway (Park et al., 2020). The ginseng leaf extract rich in ginsenosides (including ginsenoside Rg1) applied topically to mouse skin protected against UVB-induced photoaging and skin barrier dysfunction through suppression of MMP-2, MMP-9 and MMP-13 protein expressions (Hong et al., 2017). Moreover, ginsenoside Rg1 showed anti-inflammatory effects against UVB-induced glucocorticoid resistance in keratinocyte HaCaT cells via promotion of Nrf2 activity (Li et al., 2016).

The sesquiterpene lactone eupalinolide A and B from *Eupatorium lindleyanum* DC. showed inhibitory effects against UVB-induced melanogenesis, skin damage and inflammatory responses *in vitro* and mouse skin *in vivo* (Yamashita et al., 2012). Previous evidence revealed that, apart from the anti-photoaging effect, a minor ginsenoside (C-Y) having ability to induce Nrf2 activity was observed to exert whitening effects by inhibiting melanin production, tyrosinase activity in Melan-a and zebrafish embryos (Liu et al., 2019).

### Cannabidinol

Cannabidiol (CBD), the second most prevalent active ingredient in cannabis, is the non-psychoactive phytocannabinoid that has antioxidant and anti-inflammatory effects. CBD has been reported to provide photoprotective effects against UVA and UVB-induced damage to skin cells including NHDFs and keratinocyte HaCaT cells (Vacek et al., 2021). Treatment of 2D and 3D cultured fibroblasts with CBD caused a substantial attenuation in the levels of lipid peroxidation-derived aldehydes (4-hydroxynonenal (HNE), MDA and acrolein-protein adducts) in UVA (20 J/cm^2^)- and UVB (200 mJ/cm^2^)-irradiated cells (Gegotek et al., 2019). The formation of aldehyde-protein adducts induced by the highly reactive aldehydes could subsequently change the structure and/or function of several proteins including main ECM elastin in hairless mice exposed to UVA (Larroque-Cardoso et al., 2015). In addition, 4-HNE was suggested to play a role in UVA-induced fibroblast senescence in skin photoaging (Swiader et al., 2021). The compounds having abilities to neutralize aldehydes and inhibit the formation of protein adducts could thus have a beneficial role against skin photoaging.

Moreover, the analytical chemistry revealed that CBD compound could interact with the Nrf2/NF-κB transcriptional activity (Jastrzab et al., 2019). The biological effects of CBD have been found to maintain membrane integrity by preventing protein and phospholipid modifications (Atalay et al., 2020) and prevent the inflammatory responses (nuclear receptor coactivator-3 and paralemmin-3) (Atalay et al., 2021).

## Conclusion and Future Challenges: Insight Into Ethnopharmacology

Ethnopharmacology is defined as “the interdisciplinary exploration of biologically active agents traditionally employed or observed by man” (Bruhn and Rivier, 2019). Identifying the ingredients and exploring the effects of the ingredients are crucial in the study of traditional medicine. Phytochemicals are bioactive compounds in plant-based products that have been historically used to rejuvenate the skin and alleviate skin disorders. Bioactive compounds of plant origin have thus been considered as invaluable sources of potential preventive or therapeutic agents for dermatological indications due to their pharmacological activities including antioxidant, UV absorption and anti-inflammation. The phytochemicals exert antioxidant effects by directly scavenging ROS or by promoting the antioxidant defense system through activation of Nrf2 signaling. It should also be taken into consideration that while Nrf2 plays a crucial role in maintaining cellular homeostasis under stress and inflammatory conditions, several studies have discussed a detrimental aspect of Nrf2 defined as the “dark side of Nrf2” in the cancer biology as enhanced Nrf2 activity is involved in a pro-carcinogenic effect and therapeutic resistance of cancer cells (Sporn and Liby, 2012). Thus, dietary phytochemicals having the potential to provide either chemopreventive or cancer-promotive properties, depending on the stage of carcinogenesis (L Suraweera et al., 2020). This review discusses the protective role of Nrf2 against UVR-induced skin photodamage and thus application of phytochemicals acting as Nrf2 activators is regarded as a promising strategy to prevent and treat premature aging and photodamage-related skin problems. Targeting Nrf2-dependent antioxidant and cytoprotective response has been suggested to represent a promising pharmacological strategy for development of effective and safe anti-photoaging and photoprotective agents. Furthermore, the therapeutic potential of phytochemicals can be limited by their poor bioavailability and thus development of drug delivery systems (such as nano-engineered formulations) is needed to improve efficacy of promising bioactive compounds as effective photoprotective agents.

## References

[B1] Afaq F. Zaid M. A. Khan N. Dreher M. Mukhtar H. (2009). Protective Effect of Pomegranate-Derived Products on UVB-Mediated Damage in Human Reconstituted Skin. Exp. Dermatol. 18 (6), 553–561. 10.1111/j.1600-0625.2008.00829.x 19320737 PMC3004287

[B2] Ahn Y. J. Kim H. (2021). Lutein as a Modulator of Oxidative Stress-Mediated Inflammatory Diseases. Antioxidants (Basel) 10 (9). 10.3390/antiox10091448 PMC8470349 34573081

[B3] Amer R. I. Ezzat S. M. Aborehab N. M. Ragab M. F. Mohamed D. Hashad A. (2021). Downregulation of MMP1 Expression Mediates the Anti-aging Activity of Citrus Sinensis Peel Extract Nanoformulation in UV Induced Photoaging in Mice. Biomed. Pharmacother. 138, 111537. 10.1016/j.biopha.2021.111537 34311535

[B4] Angel P. Szabowski A. Schorpp-Kistner M. (2001). Function and Regulation of AP-1 Subunits in Skin Physiology and Pathology. Oncogene 20 (19), 2413–2423. 10.1038/sj.onc.1204380 11402337

[B5] Atalay S. Dobrzyńska I. Gęgotek A. Skrzydlewska E. (2020). Cannabidiol Protects Keratinocyte Cell Membranes Following Exposure to UVB and Hydrogen Peroxide. Redox Biol. 36, 101613. 10.1016/j.redox.2020.101613 32863232 PMC7327251

[B6] Atalay S. Gęgotek A. Wroński A. Domigues P. Skrzydlewska E. (2021). Therapeutic Application of Cannabidiol on UVA and UVB Irradiated Rat Skin. A Proteomic Study. J. Pharm. Biomed. Anal. 192, 113656. 10.1016/j.jpba.2020.113656 33086172

[B7] Aust O. Stahl W. Sies H. Tronnier H. Heinrich U. (2005). Supplementation with Tomato-Based Products Increases Lycopene, Phytofluene, and Phytoene Levels in Human Serum and Protects against UV-Light-Induced Erythema. Int. J. Vitam Nutr. Res. 75 (1), 54–60. 10.1024/0300-9831.75.1.54 15830922

[B8] Babich H. Schuck A. G. Weisburg J. H. Zuckerbraun H. L. (2011). Research Strategies in the Study of the Pro-oxidant Nature of Polyphenol Nutraceuticals. J. Toxicol. 2011, 467305. 10.1155/2011/467305 21776260 PMC3135211

[B9] Baird L. Yamamoto M. (2020). The Molecular Mechanisms Regulating the KEAP1-NRF2 Pathway. Mol. Cell Biol 40 (13). 10.1128/MCB.00099-20 PMC7296212 32284348

[B10] Berman B. Cockerell C. J. (2013). Pathobiology of Actinic Keratosis: Ultraviolet-dependent Keratinocyte Proliferation. J. Am. Acad. Dermatol. 68 (1 Suppl. 1), S10–S19. 10.1016/j.jaad.2012.09.053 23228301

[B11] Biniek K. Levi K. Dauskardt R. H. (2012). Solar UV Radiation Reduces the Barrier Function of Human Skin. Proc. Natl. Acad. Sci. U S A. 109 (42), 17111–17116. 10.1073/pnas.1206851109 23027968 PMC3479513

[B12] Boo Y. C. (2020a). Emerging Strategies to Protect the Skin from Ultraviolet Rays Using Plant-Derived Materials. Antioxidants (Basel) 9 (7). 10.3390/antiox9070637 PMC7402153 32708455

[B13] Boo Y. C. (2019). Human Skin Lightening Efficacy of Resveratrol and its Analogs: From *In Vitro* Studies to Cosmetic Applications. Antioxidants (Basel) 8 (9). 10.3390/antiox8090332 PMC6770230 31443469

[B14] Boo Y. C. (2020b). Natural Nrf2 Modulators for Skin Protection. Antioxidants (Basel) 9 (9). 10.3390/antiox9090812 PMC7556038 32882952

[B15] Bosch R. Philips N. Suárez-Pérez J. A. Juarranz A. Devmurari A. Chalensouk-Khaosaat J. (2015). Mechanisms of Photoaging and Cutaneous Photocarcinogenesis, and Photoprotective Strategies with Phytochemicals. Antioxidants (Basel) 4 (2), 248–268. 10.3390/antiox4020248 26783703 PMC4665475

[B16] Brand R. M. Epperly M. W. Stottlemyer J. M. Skoda E. M. Gao X. Li S. (2017). A Topical Mitochondria-Targeted Redox-Cycling Nitroxide Mitigates Oxidative Stress-Induced Skin Damage. J. Invest. Dermatol. 137 (3), 576–586. 10.1016/j.jid.2016.09.033 27794421 PMC5466072

[B17] Braun S. Hanselmann C. Gassmann M. G. auf dem Keller U. Born-Berclaz C. Chan K. (2002). auf dem Keller, UNrf2 transcription factor, a novel target of keratinocyte growth factor action which regulates gene expression and inflammation in the healing skin wound. Mol. Cell Biol 22 (15), 5492–5505. 10.1128/MCB.22.15.5492-5505.2002 12101242 PMC133949

[B18] Brem R. Macpherson P. Guven M. Karran P. (2017). Oxidative Stress Induced by UVA Photoactivation of the Tryptophan UVB Photoproduct 6-Formylindolo[3,2-B]carbazole (FICZ) Inhibits Nucleotide Excision Repair in Human Cells. Sci. Rep. 7 (1), 4310. 10.1038/s41598-017-04614-8 28655934 PMC5487344

[B19] Brenner M. Hearing V. J. (2008). The Protective Role of Melanin against UV Damage in Human Skin. Photochem. Photobiol. 84 (3), 539–549. 10.1111/j.1751-1097.2007.00226.x 18435612 PMC2671032

[B20] Bruhn J. G. Rivier L. (2019). Ethnopharmacology - A Journal, a Definition and a Society. J. Ethnopharmacol 242, 112005. 10.1016/j.jep.2019.112005 31216432

[B21] Calniquer G. Khanin M. Ovadia H. Linnewiel-Hermoni K. Stepensky D. Trachtenberg A. (2021). Combined Effects of Carotenoids and Polyphenols in Balancing the Response of Skin Cells to UV Irradiation. Molecules 26 (7). 10.3390/molecules26071931 PMC8036680 33808148

[B22] Camera E. Mastrofrancesco A. Fabbri C. Daubrawa F. Picardo M. Sies H. (2009). Astaxanthin, Canthaxanthin and Beta-Carotene Differently Affect UVA-Induced Oxidative Damage and Expression of Oxidative Stress-Responsive Enzymes. Exp. Dermatol. 18 (3), 222–231. 10.1111/j.1600-0625.2008.00790.x 18803658

[B23] Cavinato M. Waltenberger B. Baraldo G. Grade C. V. C. Stuppner H. Jansen-Dürr P. (2017). Plant Extracts and Natural Compounds Used against UVB-Induced Photoaging. Biogerontology 18 (4), 499–516. 10.1007/s10522-017-9715-7 28702744 PMC5514221

[B24] Chae J. K. Subedi L. Jeong M. Park Y. U. Kim C. Y. Kim H. (2017). Gomisin N Inhibits Melanogenesis through Regulating the PI3K/Akt and MAPK/ERK Signaling Pathways in Melanocytes. Int. J. Mol. Sci. 18 (2). 10.3390/ijms18020471 PMC5344003 28241436

[B25] Chaiprasongsuk A. Lohakul J. Soontrapa K. Sampattavanich S. Akarasereenont P. Panich U. (2017). Activation of Nrf2 Reduces UVA-Mediated MMP-1 Upregulation via MAPK/AP-1 Signaling Cascades: The Photoprotective Effects of Sulforaphane and Hispidulin. J. Pharmacol. Exp. Ther. 360 (3), 388–398. 10.1124/jpet.116.238048 28011874 PMC5325073

[B26] Chaiprasongsuk A. Onkoksoong T. Pluemsamran T. Limsaengurai S. Panich U. (2016). Photoprotection by Dietary Phenolics against Melanogenesis Induced by UVA through Nrf2-dependent Antioxidant Responses. Redox Biol. 8, 79–90. 10.1016/j.redox.2015.12.006 26765101 PMC4712325

[B27] Chang L. Karin M. (2001). Mammalian MAP Kinase Signalling Cascades. Nature 410 (6824), 37–40. 10.1038/35065000 11242034

[B28] Chang T. M. Tsen J. H. Yen H. Yang T. Y. Huang H. C. (2017). Extract from Periostracum Cicadae Inhibits Oxidative Stress and Inflammation Induced by Ultraviolet B Irradiation on HaCaT Keratinocytes. Evid. Based Complement. Alternat Med. 2017, 8325049. 10.1155/2017/8325049 28465707 PMC5390570

[B29] Chen F. Tang Y. Sun Y. Veeraraghavan V. P. Mohan S. K. Cui C. (2019). 6-shogaol, a Active Constiuents of Ginger Prevents UVB Radiation Mediated Inflammation and Oxidative Stress through Modulating NrF2 Signaling in Human Epidermal Keratinocytes (HaCaT Cells). J. Photochem. Photobiol. B 197, 111518. 10.1016/j.jphotobiol.2019.111518 31202076

[B30] Chen L. G. Chang W. L. Lee C. J. Lee L. T. Shih C. M. Wang C. C. (2009). Melanogenesis Inhibition by Gallotannins from Chinese Galls in B16 Mouse Melanoma Cells. Biol. Pharm. Bull. 32 (8), 1447–1452. 10.1248/bpb.32.1447 19652388

[B31] Chen L. Y. Cheng H. L. Kuan Y. H. Liang T. J. Chao Y. Y. Lin H. C. (2021a). Therapeutic Potential of Luteolin on Impaired Wound Healing in Streptozotocin-Induced Rats. Biomedicines 9 (7). 10.3390/biomedicines9070761 PMC8301369 34209369

[B32] Chen S. J. Hseu Y. C. Gowrisankar Y. V. Chung Y. T. Zhang Y. Z. Way T. D. (2021b). The Anti-melanogenic Effects of 3-O-Ethyl Ascorbic Acid via Nrf2-Mediated α-MSH Inhibition in UVA-Irradiated Keratinocytes and Autophagy Induction in Melanocytes. Free Radic. Biol. Med. 173, 151–169. 10.1016/j.freeradbiomed.2021.07.030 34314818

[B33] Chen X. Liu Y. Zhu J. Lei S. Dong Y. Li L. (2016). GSK-3β Downregulates Nrf2 in Cultured Cortical Neurons and in a Rat Model of Cerebral Ischemia-Reperfusion. Sci. Rep. 6, 20196. 10.1038/srep20196 26838164 PMC4738318

[B34] Cho B. O. Che D. N. Shin J. Y. Kang H. J. Jang S. I. (2018). Ameliorative Effects of Fruit Stem Extract from Muscat Bailey A against Chronic UV-Induced Skin Damage in BALB/c Mice. Biomed. Pharmacother. 97, 1680–1688. 10.1016/j.biopha.2017.12.003 29793331

[B35] Choi S. J. Lee S. N. Kim K. Joo da. H. Shin S. Lee J. (2016). Biological Effects of Rutin on Skin Aging. Int. J. Mol. Med. 38 (1), 357–363. 10.3892/ijmm.2016.2604 27220601

[B36] Ciganović P. Jakimiuk K. Tomczyk M. Zovko Končić M. (2019). Glycerolic Licorice Extracts as Active Cosmeceutical Ingredients: Extraction Optimization, Chemical Characterization, and Biological Activity. Antioxidants 8 (10), 445. 10.3390/antiox8100445 PMC6826613 31581512

[B37] Cleasby A. Yon J. Day P. J. Richardson C. Tickle I. J. Williams P. A. (2014). Structure of the BTB Domain of Keap1 and its Interaction with the Triterpenoid Antagonist CDDO. PLoS One 9 (6), e98896. 10.1371/journal.pone.0098896 24896564 PMC4045772

[B38] Cooperstone J. L. Tober K. L. Riedl K. M. Teegarden M. D. Cichon M. J. Francis D. M. (2017). Tomatoes Protect against Development of UV-Induced Keratinocyte Carcinoma via Metabolomic Alterations. Sci. Rep. 7 (1), 5106. 10.1038/s41598-017-05568-7 28698610 PMC5506060

[B39] Cui B. Wang Y. Jin J. Yang Z. Guo R. Li X. (2022). Resveratrol Treats UVB-Induced Photoaging by Anti-MMP Expression, through Anti-inflammatory, Antioxidant, and Antiapoptotic Properties, and Treats Photoaging by Upregulating VEGF-B Expression. Oxid Med. Cell Longev 2022, 6037303. 10.1155/2022/6037303 35028009 PMC8752231

[B40] D'Orazio J. Jarrett S. Amaro-Ortiz A. Scott T. (2013). UV Radiation and the Skin. Int. J. Mol. Sci. 14 (6), 12222–12248. 10.3390/ijms140612222 23749111 PMC3709783

[B41] Darawsha A. Trachtenberg A. Levy J. Sharoni Y. (2021). The Protective Effect of Carotenoids, Polyphenols, and Estradiol on Dermal Fibroblasts under Oxidative Stress. Antioxidants (Basel) 10 (12). 10.3390/antiox10122023 PMC8698602 34943127

[B42] Dayalan Naidu S. Dinkova-Kostova A. T. (2020). KEAP1, a Cysteine-Based Sensor and a Drug Target for the Prevention and Treatment of Chronic Disease. Open Biol. 10 (6), 200105. 10.1098/rsob.200105 32574549 PMC7333886

[B43] Dayalan Naidu S. Muramatsu A. Saito R. Asami S. Honda T. Hosoya T. (2018). C151 in KEAP1 Is the Main Cysteine Sensor for the Cyanoenone Class of NRF2 Activators, Irrespective of Molecular Size or Shape. Sci. Rep. 8 (1), 8037. 10.1038/s41598-018-26269-9 29795117 PMC5966396

[B44] Denat L. Kadekaro A. L. Marrot L. Leachman S. A. Abdel-Malek Z. A. (2014). Melanocytes as Instigators and Victims of Oxidative Stress. J. Invest. Dermatol. 134 (6), 1512–1518. 10.1038/jid.2014.65 24573173 PMC4418514

[B45] Deshmukh P. Unni S. Krishnappa G. Padmanabhan B. (2017). The Keap1-Nrf2 Pathway: Promising Therapeutic Target to Counteract ROS-Mediated Damage in Cancers and Neurodegenerative Diseases. Biophys. Rev. 9 (1), 41–56. 10.1007/s12551-016-0244-4 28510041 PMC5425799

[B46] Didier C. Kerblat I. Drouet C. Favier A. Béani J. C. Richard M. J. (2001). Induction of Thioredoxin by Ultraviolet-A Radiation Prevents Oxidative-Mediated Cell Death in Human Skin Fibroblasts. Free Radic. Biol. Med. 31 (5), 585–598. 10.1016/s0891-5849(01)00617-7 11522443

[B47] Dinkova-Kostova A. T. Talalay P. (2008). Direct and Indirect Antioxidant Properties of Inducers of Cytoprotective Proteins. Mol. Nutr. Food Res. 52 Suppl 1 (Suppl. 1), S128–S138. 10.1002/mnfr.200700195 18327872

[B48] Dissemond J. Schneider L. A. Brenneisen P. Briviba K. Wenk J. Wlaschek M. (2003). Protective and Determining Factors for the Overall Lipid Peroxidation in Ultraviolet A1-Irradiated Fibroblasts: *In Vitro* and *In Vivo* Investigations. Br. J. Dermatol. 149 (2), 341–349. 10.1046/j.1365-2133.2003.05457.x 12932241

[B49] Du D. Yao L. Zhang R. Shi N. Shen Y. Yang X. (2018). Protective Effects of Flavonoids from Coreopsis Tinctoria Nutt. On Experimental Acute Pancreatitis via Nrf-2/ARE-Mediated Antioxidant Pathways. J. Ethnopharmacol 224, 261–272. 10.1016/j.jep.2018.06.003 29870787

[B50] Dunaway S. Odin R. Zhou L. Ji L. Zhang Y. Kadekaro A. L. (2018). Natural Antioxidants: Multiple Mechanisms to Protect Skin from Solar Radiation. Front. Pharmacol. 9, 392. 10.3389/fphar.2018.00392 29740318 PMC5928335

[B51] Egawa G. Kabashima K. (2018). Barrier Dysfunction in the Skin Allergy. Allergol. Int. 67 (1), 3–11. 10.1016/j.alit.2017.10.002 29153780

[B52] Espinosa-Diez C. Miguel V. Mennerich D. Kietzmann T. Sánchez-Pérez P. Cadenas S. (2015). Antioxidant Responses and Cellular Adjustments to Oxidative Stress. Redox Biol. 6, 183–197. 10.1016/j.redox.2015.07.008 26233704 PMC4534574

[B53] Fanjul-Fernández M. Folgueras A. R. Cabrera S. López-Otín C. (2010). Matrix Metalloproteinases: Evolution, Gene Regulation and Functional Analysis in Mouse Models. Biochim. Biophys. Acta 1803 (1), 3–19. 10.1016/j.bbamcr.2009.07.004 19631700

[B54] Gao W. Lin P. Hwang E. Wang Y. Yan Z. Ngo H. T. T. (2018a). Pterocarpus Santalinus L. Regulated Ultraviolet B Irradiation-Induced Procollagen Reduction and Matrix Metalloproteinases Expression through Activation of TGF-β/Smad and Inhibition of the MAPK/AP-1 Pathway in Normal Human Dermal Fibroblasts. Photochem. Photobiol. 94 (1), 139–149. 10.1111/php.12835 28858391

[B55] Gao W. Wang Y.-s. Hwang E. Lin P. Bae J. Seo S. A. (2018b). Rubus Idaeus L. (Red Raspberry) Blocks UVB-Induced MMP Production and Promotes Type I Procollagen Synthesis via Inhibition of MAPK/AP-1, NF-Κβ and Stimulation of TGF-β/Smad, Nrf2 in normal Human Dermal Fibroblasts. J. Photochem. Photobiol. B: Biol. 185, 241–253. 10.1016/j.jphotobiol.2018.06.007 29966991

[B56] Garg C. Sharma H. Garg M. (2020). Skin Photo-protection with Phytochemicals against Photo-Oxidative Stress, Photo-Carcinogenesis, Signal Transduction Pathways and Extracellular Matrix Remodeling-An Overview. Ageing Res. Rev. 62, 101127. 10.1016/j.arr.2020.101127 32721499

[B57] Gęgotek A. Atalay S. Domingues P. Skrzydlewska E. (2019). The Differences in the Proteome Profile of Cannabidiol-Treated Skin Fibroblasts Following UVA or UVB Irradiation in 2D and 3D Cell Cultures. Cells 8 (9). 10.3390/cells8090995 PMC6770406 31466340

[B58] Gęgotek A. Domingues P. Skrzydlewska E. (2018). Proteins Involved in the Antioxidant and Inflammatory Response in Rutin-Treated Human Skin Fibroblasts Exposed to UVA or UVB Irradiation. J. Dermatol. Sci. 90 (3), 241–252. 10.1016/j.jdermsci.2018.02.002 29455850

[B59] Gęgotek A. Rybałtowska-Kawałko P. Skrzydlewska E. (2017). Rutin as a Mediator of Lipid Metabolism and Cellular Signaling Pathways Interactions in Fibroblasts Altered by UVA and UVB Radiation. Oxidative Med. Cell Longevity 2017, 1–20. 10.1155/2017/4721352 PMC5266866 28168010

[B60] Gęgotek A. Skrzydlewska E. (2015). The Role of Transcription Factor Nrf2 in Skin Cells Metabolism. Arch. Dermatol. Res. 307 (5), 385–396. 10.1007/s00403-015-1554-2 25708189 PMC4469773

[B61] Greenlee K. J. Werb Z. Kheradmand F. (2007). Matrix Metalloproteinases in Lung: Multiple, Multifarious, and Multifaceted. Physiol. Rev. 87 (1), 69–98. 10.1152/physrev.00022.2006 17237343 PMC2656382

[B62] Gruber F. Mayer H. Lengauer B. Mlitz V. Sanders J. M. Kadl A. (2010). NF-E2-related Factor 2 Regulates the Stress Response to UVA-1-Oxidized Phospholipids in Skin Cells. FASEB J. 24 (1), 39–48. 10.1096/fj.09-133520 19720622 PMC2797031

[B63] Gunaseelan S. Balupillai A. Govindasamy K. Ramasamy K. Muthusamy G. Shanmugam M. (2017). Linalool Prevents Oxidative Stress Activated Protein Kinases in Single UVB-Exposed Human Skin Cells. PLoS One 12 (5), e0176699. 10.1371/journal.pone.0176699 28467450 PMC5415184

[B64] Halliday G. M. Lyons J. G. (2008). Inflammatory Doses of UV May Not Be Necessary for Skin Carcinogenesis. Photochem. Photobiol. 84 (2), 272–283. 10.1111/j.1751-1097.2007.00247.x 18353168

[B65] Hano C. Tungmunnithum D. (2020). Plant Polyphenols, More Than Just Simple Natural Antioxidants: Oxidative Stress, Aging and Age-Related Diseases. Medicines (Basel) 7 (5). 10.3390/medicines7050026 PMC7281114 32397520

[B66] Hayashi R. Himori N. Taguchi K. Ishikawa Y. Uesugi K. Ito M. (2013). The Role of the Nrf2-Mediated Defense System in Corneal Epithelial Wound Healing. Free Radic. Biol. Med. 61, 333–342. 10.1016/j.freeradbiomed.2013.04.008 23587556

[B67] He F. Ru X. Wen T. (2020). NRF2, a Transcription Factor for Stress Response and beyond. Int. J. Mol. Sci. 21 (13). 10.3390/ijms21134777 PMC7369905 32640524

[B68] Heinrich U. Neukam K. Tronnier H. Sies H. Stahl W. (2006). Long-term Ingestion of High Flavanol cocoa Provides Photoprotection against UV-Induced Erythema and Improves Skin Condition in Women. J. Nutr. 136 (6), 1565–1569. 10.1093/jn/136.6.1565 16702322

[B69] Hong Y. H. Lee H. S. Jung E. Y. Han S. H. Park Y. Suh H. J. (2017). Photoprotective Effects of Topical Ginseng Leaf Extract Using Ultraflo L against UVB-Induced Skin Damage in Hairless Mice. J. Ginseng Res. 41 (4), 456–462. 10.1016/j.jgr.2016.07.007 29021691 PMC5628359

[B70] Hseu Y. C. Chang C. T. Gowrisankar Y. V. Chen X. Z. Lin H. C. Yen H. R. (2019). Zerumbone Exhibits Antiphotoaging and Dermatoprotective Properties in Ultraviolet A-Irradiated Human Skin Fibroblast Cells via the Activation of Nrf2/ARE Defensive Pathway. Oxid Med. Cell Longev 2019, 4098674. 10.1155/2019/4098674 31814875 PMC6878809

[B71] Hseu Y. C. Chen X. Z. Vudhya Gowrisankar Y. Yen H. R. Chuang J. Y. Yang H. L. (2020). The Skin-Whitening Effects of Ectoine via the Suppression of α-MSH-Stimulated Melanogenesis and the Activation of Antioxidant Nrf2 Pathways in UVA-Irradiated Keratinocytes. Antioxidants (Basel) 9 (1). 10.3390/antiox9010063 PMC7022695 31936771

[B72] Hseu Y. C. Chou C. W. Senthil Kumar K. J. Fu K. T. Wang H. M. Hsu L. S. (2012). Ellagic Acid Protects Human Keratinocyte (HaCaT) Cells against UVA-Induced Oxidative Stress and Apoptosis through the Upregulation of the HO-1 and Nrf-2 Antioxidant Genes. Food Chem. Toxicol. 50 (5), 1245–1255. 10.1016/j.fct.2012.02.020 22386815

[B73] Hseu Y. C. Vudhya Gowrisankar Y. Wang L. W. Zhang Y. Z. Chen X. Z. Huang P. J. (2021). The *In Vitro* and *In Vivo* Depigmenting Activity of Pterostilbene through Induction of Autophagy in Melanocytes and Inhibition of UVA-Irradiated α-MSH in Keratinocytes via Nrf2-Mediated Antioxidant Pathways. Redox Biol. 44, 102007. 10.1016/j.redox.2021.102007 34049220 PMC8167190

[B74] Huang C.-Y. Liu I.-H. Huang X.-Z. Chen H.-J. Chang S.-T. Chang M.-L. (2021). Antimelanogenesis Effects of Leaf Extract and Phytochemicals from Ceylon Olive (Elaeocarpus Serratus) in Zebrafish Model. Pharmaceutics 13 (7), 1059. 10.3390/pharmaceutics13071059 34371750 PMC8309042

[B75] Hwang E. Gao W. Xiao Y. K. Ngo H. T. T. Yi T. H. (2019). Helianthus Annuus L. Flower Prevents UVB-Induced Photodamage in Human Dermal Fibroblasts by Regulating the MAPK/AP-1, NFAT, and Nrf2 Signaling Pathways. J. Cell Biochem 120 (1), 601–612. 10.1002/jcb.27417 30195253

[B76] Hwang E. Ngo H. T. T. Seo S. A. Park B. Zhang M. Yi T. H. (2018). Protective Effect of Dietary Alchemilla Mollis on UVB-Irradiated Premature Skin Aging through Regulation of Transcription Factor NFATc1 and Nrf2/ARE Pathways. Phytomedicine 39, 125–136. 10.1016/j.phymed.2017.12.025 29433674

[B77] Ikehata H. Yamamoto M. (2018). Roles of the KEAP1-NRF2 System in Mammalian Skin Exposed to UV Radiation. Toxicol. Appl. Pharmacol. 360, 69–77. 10.1016/j.taap.2018.09.038 30268578

[B78] Jastrząb A. Gęgotek A. Skrzydlewska E. (2019). Cannabidiol Regulates the Expression of Keratinocyte Proteins Involved in the Inflammation Process through Transcriptional Regulation. Cells 8 (8), 827. 10.3390/cells8080827 PMC6721680 31382646

[B79] Jeayeng S. Wongkajornsilp A. Slominski A. T. Jirawatnotai S. Sampattavanich S. Panich U. (2017). Nrf2 in Keratinocytes Modulates UVB-Induced DNA Damage and Apoptosis in Melanocytes through MAPK Signaling. Free Radic. Biol. Med. 108, 918–928. 10.1016/j.freeradbiomed.2017.05.009 28495448 PMC5546090

[B80] Jian Z. Li K. Liu L. Zhang Y. Zhou Z. Li C. (2011). Heme Oxygenase-1 Protects Human Melanocytes from H2O2-Induced Oxidative Stress via the Nrf2-ARE Pathway. J. Invest. Dermatol. 131 (7), 1420–1427. 10.1038/jid.2011.56 21412259

[B81] Jo D. J. Seok J. K. Kim S. Y. Park W. Baek J. H. Kim Y. M. (2018). Human Skin-Depigmenting Effects of Resveratryl Triglycolate, a Hybrid Compound of Resveratrol and Glycolic Acid. Int. J. Cosmet. Sci. 40, 256–262. 10.1111/ics.12458 29663438

[B82] Johnsson P. (2004). Phenolic Compounds in Flaxseed Chromatographic and Spectroscopic Analyses of Glucosidic Conjugates. Uppsala, Sweden: Swedish University of Agricultural Sciences.

[B83] Kanlayavattanakul M. Chongnativisit W. Chaikul P. Lourith N. (2020). Phenolic-rich Pomegranate Peel Extract: *In Vitro*, Cellular, and *In Vivo* Activities for Skin Hyperpigmentation Treatment. Planta Med. 86 (11), 749–759. 10.1055/a-1170-7785 32428937

[B84] Kannan S. Jaiswal A. K. (2006). Low and High Dose UVB Regulation of Transcription Factor NF-E2-Related Factor 2. Cancer Res. 66 (17), 8421–8429. 10.1158/0008-5472.CAN-06-1181 16951152

[B85] Karin M. (1995). The Regulation of AP-1 Activity by Mitogen-Activated Protein Kinases. J. Biol. Chem. 270 (28), 16483–16486. 10.1074/jbc.270.28.16483 7622446

[B86] Kawachi Y. Xu X. Taguchi S. Sakurai H. Nakamura Y. Ishii Y. (2008). Attenuation of UVB-Induced Sunburn Reaction and Oxidative DNA Damage with No Alterations in UVB-Induced Skin Carcinogenesis in Nrf2 Gene-Deficient Mice. J. Invest. Dermatol. 128 (7), 1773–1779. 10.1038/sj.jid.5701245 18200051

[B87] Kerns M. L. Hakim J. M. Lu R. G. Guo Y. Berroth A. Kaspar R. L. (2016). Oxidative Stress and Dysfunctional NRF2 Underlie Pachyonychia Congenita Phenotypes. J. Clin. Invest. 126 (6), 2356–2366. 10.1172/JCI84870 27183391 PMC4887188

[B88] Kim A. J. Park J. E. Cho Y. H. Lim D. S. Lee J. S. (2021). Effect of 7-Methylsulfinylheptyl Isothiocyanate on the Inhibition of Melanogenesis in B16-F1 Cells. Life (Basel) 11 (2). 10.3390/life11020162 PMC7923422 33672463

[B89] Kim J. Kim J. Shim J. Lee C. Y. Lee K. W. Lee H. J. (2014). Cocoa Phytochemicals: Recent Advances in Molecular Mechanisms on Health. Crit. Rev. Food Sci. Nutr. 54 (11), 1458–1472. 10.1080/10408398.2011.641041 24580540

[B90] Kim J. Oh J. Averilla J. N. Kim H. J. Kim J. S. Kim J. S. (2019). Grape Peel Extract and Resveratrol Inhibit Wrinkle Formation in Mice Model through Activation of Nrf2/HO-1 Signaling Pathway. J. Food Sci. 84 (6), 1600–1608. 10.1111/1750-3841.14643 31132143

[B91] Kim J. M. Noh E. M. Kwon K. B. Hwang B. M. Hwang J. K. You Y. O. (2013). Dihydroavenanthramide D Prevents UV-Irradiated Generation of Reactive Oxygen Species and Expression of Matrix Metalloproteinase-1 and -3 in Human Dermal Fibroblasts. Exp. Dermatol. 22 (11), 759–761. 10.1111/exd.12243 24103002 PMC4251632

[B92] Kim M. Park Y. G. Lee H. J. Lim S. J. Nho C. W. (2015). Youngiasides A and C Isolated from Youngia Denticulatum Inhibit UVB-Induced MMP Expression and Promote Type I Procollagen Production via Repression of MAPK/AP-1/NF-κB and Activation of AMPK/Nrf2 in HaCaT Cells and Human Dermal Fibroblasts. J. Agric. Food Chem. 63 (22), 5428–5438. 10.1021/acs.jafc.5b00467 25994852

[B93] Kishimoto Y. Tani M. Uto-Kondo H. Iizuka M. Saita E. Sone H. (2010). Astaxanthin Suppresses Scavenger Receptor Expression and Matrix Metalloproteinase Activity in Macrophages. Eur. J. Nutr. 49 (2), 119–126. 10.1007/s00394-009-0056-4 19784539

[B94] Kleszczyński K. Ernst I. M. Wagner A. E. Kruse N. Zillikens D. Rimbach G. (2013). Sulforaphane and Phenylethyl Isothiocyanate Protect Human Skin against UVR-Induced Oxidative Stress and Apoptosis: Role of Nrf2-dependent Gene Expression and Antioxidant Enzymes. Pharmacol. Res. 78, 28–40. 10.1016/j.phrs.2013.09.009 24121007

[B95] Ko H. J. Kim J. H. Lee G. S. Shin T. (2020). Sulforaphane Controls the Release of Paracrine Factors by Keratinocytes and Thus Mitigates Particulate Matter-Induced Premature Skin Aging by Suppressing Melanogenesis and Maintaining Collagen Homeostasis. Phytomedicine 77, 153276. 10.1016/j.phymed.2020.153276 32659677

[B96] Kohandel Z. Farkhondeh T. Aschner M. Pourbagher-Shahri A. M. Samarghandian S. (2022). Anti-inflammatory Action of Astaxanthin and its Use in the Treatment of Various Diseases. Biomed. Pharmacother. 145, 112179. 10.1016/j.biopha.2021.112179 34736076

[B97] Kohandel Z. Farkhondeh T. Aschner M. Samarghandian S. (2021). Nrf2 a Molecular Therapeutic Target for Astaxanthin. Biomed. Pharmacother. 137, 111374. 10.1016/j.biopha.2021.111374 33761600

[B98] Kong A. N. Owuor E. Yu R. Hebbar V. Chen C. Hu R. (2001). Induction of Xenobiotic Enzymes by the MAP Kinase Pathway and the Antioxidant or Electrophile Response Element (ARE/EpRE). Drug Metab. Rev. 33 (3-4), 255–271. 10.1081/dmr-120000652 11768769

[B99] Kuwano T. Watanabe M. Kagawa D. Murase T. (2015). Hydrolyzed Methylhesperidin Induces Antioxidant Enzyme Expression via the Nrf2-ARE Pathway in Normal Human Epidermal Keratinocytes. J. Agric. Food Chem. 63 (36), 7937–7944. 10.1021/acs.jafc.5b01992 26313892

[B100] Kypriotou M. Huber M. Hohl D. (2012). The Human Epidermal Differentiation Complex: Cornified Envelope Precursors, S100 Proteins and the 'fused Genes' Family. Exp. Dermatol. 21 (9), 643–649. 10.1111/j.1600-0625.2012.01472.x 22507538

[B101] L Suraweera T. Rupasinghe H. P. V. Dellaire G. Xu Z. (2020). Regulation of Nrf2/ARE Pathway by Dietary Flavonoids: A Friend or Foe for Cancer Management? Antioxidants (Basel) 9 (10). 10.3390/antiox9100973 PMC7600646 33050575

[B102] Larroque-Cardoso P. Camaré C. Nadal-Wollbold F. Grazide M. H. Pucelle M. Garoby-Salom S. (2015). Elastin Modification by 4-Hydroxynonenal in Hairless Mice Exposed to UV-A. Role in Photoaging and Actinic Elastosis. J. Invest. Dermatol. 135 (7), 1873–1881. 10.1038/jid.2015.84 25739050

[B103] Latronico T. Larocca M. Milella S. Fasano A. Rossano R. Liuzzi G. M. (2021). Neuroprotective Potential of Isothiocyanates in an *In Vitro* Model of Neuroinflammation. Inflammopharmacology 29 (2), 561–571. 10.1007/s10787-020-00772-w 33196947 PMC7997826

[B104] Lee E.-J. Zheng M. Craft C. M. Jeong S. (2021). Matrix Metalloproteinase-9 (MMP-9) and Tissue Inhibitor of Metalloproteinases 1 (TIMP-1) Are Localized in the Nucleus of Retinal Müller Glial Cells and Modulated by Cytokines and Oxidative Stress. PLoS One 16 (7), e0253915. 10.1371/journal.pone.0253915 34270579 PMC8284794

[B105] Lee T. H. Seo J. O. Baek S. H. Kim S. Y. (2014). Inhibitory Effects of Resveratrol on Melanin Synthesis in Ultraviolet B-Induced Pigmentation in Guinea Pig Skin. Biomol. Ther. (Seoul) 22 (1), 35–40. 10.4062/biomolther.2013.081 24596619 PMC3936427

[B106] Lee Y. M. Seon M. R. Cho H. J. Kim J. S. Park J. H. (2009). Benzyl Isothiocyanate Exhibits Anti-inflammatory Effects in Murine Macrophages and in Mouse Skin. J. Mol. Med. (Berl) 87 (12), 1251–1261. 10.1007/s00109-009-0532-6 19760383

[B107] Lephart E. D. (2016). Skin Aging and Oxidative Stress: Equol's Anti-aging Effects via Biochemical and Molecular Mechanisms. Ageing Res. Rev. 31, 36–54. 10.1016/j.arr.2016.08.001 27521253

[B108] Li H. Jiang N. Liang B. Liu Q. Zhang E. Peng L. (2017a). Pterostilbene Protects against UVB-Induced Photo-Damage through a Phosphatidylinositol-3-kinase-dependent Nrf2/ARE Pathway in Human Keratinocytes. Redox Rep. 22 (6), 501–507. 10.1080/13510002.2017.1329917 28532341 PMC8900625

[B109] Li H. Li Z. Peng L. Jiang N. Liu Q. Zhang E. (2017b). Lycium Barbarum Polysaccharide Protects Human Keratinocytes against UVB-Induced Photo-Damage. Free Radic. Res. 51 (2), 200–210. 10.1080/10715762.2017.1294755 28287048

[B110] Li J. Liu D. Wu J. Zhang D. Cheng B. Zhang Y. (2016). Ginsenoside Rg1 Attenuates Ultraviolet B-Induced Glucocortisides Resistance in Keratinocytes via Nrf2/HDAC2 Signalling. Sci. Rep. 6, 39336. 10.1038/srep39336 27982079 PMC5159887

[B111] Li L. Hwang E. Ngo H. T. T. Lin P. Gao W. Liu Y. (2018). Antiphotoaging Effect of Prunus Yeonesis Blossom Extract via Inhibition of MAPK/AP-1 and Regulation of the TGF-βI/Smad and Nrf2/ARE Signaling Pathways. Photochem. Photobiol. 94 (4), 725–732. 10.1111/php.12894 29421853

[B112] Li L. Ngo H. T. T. Hwang E. Wei X. Liu Y. Liu J. (2019). Conditioned Medium from Human Adipose-Derived Mesenchymal Stem Cell Culture Prevents UVB-Induced Skin Aging in Human Keratinocytes and Dermal Fibroblasts. Int. J. Mol. Sci. 21 (1). 10.3390/ijms21010049 PMC6981944 31861704

[B113] Liebel F. Kaur S. Ruvolo E. Kollias N. Southall M. D. (2012). Irradiation of Skin with Visible Light Induces Reactive Oxygen Species and Matrix-Degrading Enzymes. J. Invest. Dermatol. 132 (7), 1901–1907. 10.1038/jid.2011.476 22318388

[B114] Lin J. Y. Fisher D. E. (2007). Melanocyte Biology and Skin Pigmentation. Nature 445 (7130), 843–850. 10.1038/nature05660 17314970

[B115] Lin Z. Marepally S. R. Kim T. K. Janjetovic Z. Oak A. S. Postlethwaite A. E. (2016). Design, Synthesis and Biological Activities of Novel Gemini 20S-Hydroxyvitamin D3 Analogs. Anticancer Res. 36 (3), 877–886. 26976974 PMC5363177

[B116] Liu C. Vojnovic D. Kochevar I. E. Jurkunas U. V. (2016). UV-A Irradiation Activates Nrf2-Regulated Antioxidant Defense and Induces p53/Caspase3-dependent Apoptosis in Corneal Endothelial Cells. Invest. Ophthalmol. Vis. Sci. 57 (4), 2319–2327. 10.1167/iovs.16-19097 27127932 PMC4855825

[B117] Liu J. Liu Y. He X. Teng B. McRae J. M. (2021). Valonea Tannin: Tyrosinase Inhibition Activity, Structural Elucidation and Insights into the Inhibition Mechanism. Molecules 26 (9), 2747. 10.3390/molecules26092747 34067030 PMC8125085

[B118] Liu X. Y. Hwang E. Park B. Ngo H. T. T. Xiao Y. K. Yi T. H. (2018). Ginsenoside C-Mx Isolated from Notoginseng Stem-Leaf Ginsenosides Attenuates Ultraviolet B-Mediated Photoaging in Human Dermal Fibroblasts. Photochem. Photobiol. 94 (5), 1040–1048. 10.1111/php.12940 29779217

[B119] Liu X. Y. Li H. Hwang E. Park B. Xiao Y. K. Liu S. (2022). Chemical Distance Measurement and System Pharmacology Approach Uncover the Novel Protective Effects of Biotransformed Ginsenoside C-Mc against UVB-Irradiated Photoaging. Oxid Med. Cell Longev 2022, 4691576. 10.1155/2022/4691576 35186187 PMC8850047

[B120] Liu X. Y. Xiao Y. K. Hwang E. Haeng J. J. Yi T. H. (2019). Antiphotoaging and Antimelanogenesis Properties of Ginsenoside C-Y, a Ginsenoside Rb2 Metabolite from American Ginseng PDD-Ginsenoside. Photochem. Photobiol. 95 (6), 1412–1423. 10.1111/php.13116 31074886

[B121] Lohakul J. Chaiprasongsuk A. Jeayeng S. Saelim M. Muanjumpon P. Thanachaiphiwat S. (2021a). The Protective Effect of Polyherbal Formulation, Harak Formula, on UVA-Induced Photoaging of Human Dermal Fibroblasts and Mouse Skin via Promoting Nrf2-Regulated Antioxidant Defense. Front. Pharmacol. 12, 649820. 10.3389/fphar.2021.649820 33912060 PMC8072377

[B122] Lohakul J. Jeayeng S. Chaiprasongsuk A. Torregrossa R. Wood M. E. Saelim M. (2021b). Mitochondria-Targeted Hydrogen Sulfide Delivery Molecules Protect against UVA-Induced Photoaging in Human Dermal Fibroblasts, and in Mouse Skin *In Vivo* . Antioxid. Redox Signaling 2021. 10.1089/ars.2020.8255 34235951

[B123] López-Camarillo C. Ocampo E. A. Casamichana M. L. Pérez-Plasencia C. Alvarez-Sánchez E. Marchat L. A. (2012). Protein Kinases and Transcription Factors Activation in Response to UV-Radiation of Skin: Implications for Carcinogenesis. Int. J. Mol. Sci. 13 (1), 142–172. 10.3390/ijms13010142 22312244 PMC3269678

[B124] Lü J. Kundrát M. Shen C. (2016). New Material of the Pterosaur Gladocephaloideus Lü et al., 2012 from the Early Cretaceous of Liaoning Province, China, with Comments on Its Systematic Position. PLoS One 11 (6), e0154888. 10.1371/journal.pone.0154888 27249021 PMC4889066

[B125] Lu Y. F. Tonissen K. Di Trapani G. (2021). Modulating Skin Colour: Role of the Thioredoxin and Glutathione Systems in Regulating Melanogenesis. Biosci. Rep. 41 (5). 10.1042/BSR20210427 PMC8112849 33871027

[B126] Luo J. F. Shen X. Y. Lio C. K. Dai Y. Cheng C. S. Liu J. X. (2018). Activation of Nrf2/HO-1 Pathway by Nardochinoid C Inhibits Inflammation and Oxidative Stress in Lipopolysaccharide-Stimulated Macrophages. Front. Pharmacol. 9, 911. 10.3389/fphar.2018.00911 30233360 PMC6131578

[B127] Lv J. Fu Y. Cao Y. Jiang S. Yang Y. Song G. (2020). Isoliquiritigenin Inhibits Melanogenesis, Melanocyte Dendricity and Melanosome Transport by Regulating ERK-Mediated MITF Degradation. Exp. Dermatol. 29 (2), 149–157. 10.1111/exd.14066 31785162

[B128] Ma Q. Liu Q. Yuan L. Zhuang Y. (2018). Protective Effects of LSGYGP from Fish Skin Gelatin Hydrolysates on UVB-Induced MEFs by Regulation of Oxidative Stress and Matrix Metalloproteinase Activity. Nutrients 10 (4). 10.3390/nu10040420 PMC5946205 29597313

[B129] Ma Q. (2013). Role of Nrf2 in Oxidative Stress and Toxicity. Annu. Rev. Pharmacol. Toxicol. 53, 401–426. 10.1146/annurev-pharmtox-011112-140320 23294312 PMC4680839

[B130] Mackay A. R. Ballin M. Pelina M. D. Farina A. R. Nason A. M. Hartzler J. L. (1992). Effect of Phorbol Ester and Cytokines on Matrix Metalloproteinase and Tissue Inhibitor of Metalloproteinase Expression in Tumor and normal Cell Lines. Invasion Metastasis 12 (3-4), 168–184. 1284126

[B131] Majeed M. Majeed S. Jain R. Mundkur L. Rajalakshmi H. R. Lad P. S. (2020). An Open-Label Single-Arm, Monocentric Study Assessing the Efficacy and Safety of Natural Pterostilbene (Pterocarpus Marsupium) for Skin Brightening and Antiaging Effects. Clin. Cosmet. Investig. Dermatol. 13, 105–116. 10.2147/CCID.S238358 PMC6999773 32099438

[B132] Maleki S. J. Crespo J. F. Cabanillas B. (2019). Anti-inflammatory Effects of Flavonoids. Food Chem. 299, 125124. 10.1016/j.foodchem.2019.125124 31288163

[B133] McBride K. Nemer M. (1998). The C-Terminal Domain of C-Fos Is Required for Activation of an AP-1 Site Specific for Jun-Fos Heterodimers. Mol. Cell Biol 18 (9), 5073–5081. 10.1128/MCB.18.9.5073 9710591 PMC109092

[B134] Milani A. Basirnejad M. Shahbazi S. Bolhassani A. (2017). Carotenoids: Biochemistry, Pharmacology and Treatment. Br. J. Pharmacol. 174 (11), 1290–1324. 10.1111/bph.13625 27638711 PMC5429337

[B135] Misawa E. Tanaka M. Saito M. Nabeshima K. Yao R. Yamauchi K. (2017). Protective Effects of Aloe Sterols against UVB-Induced Photoaging in Hairless Mice. Photodermatol. Photoimmunol Photomed. 33 (2), 101–111. 10.1111/phpp.12286 27995657

[B136] Narayanan D. L. Saladi R. N. Fox J. L. (2010). Ultraviolet Radiation and Skin Cancer. Int. J. Dermatol. 49 (9), 978–986. 10.1111/j.1365-4632.2010.04474.x 20883261

[B137] Nguyen T. Nioi P. Pickett C. B. (2009). The Nrf2-Antioxidant Response Element Signaling Pathway and its Activation by Oxidative Stress. J. Biol. Chem. 284 (20), 13291–13295. 10.1074/jbc.R900010200 19182219 PMC2679427

[B138] Ni H. Jin W. Zhu T. Wang J. Yuan B. Jiang J. (2015). Curcumin Modulates TLR4/NF-κB Inflammatory Signaling Pathway Following Traumatic Spinal Cord Injury in Rats. J. Spinal Cord Med. 38 (2), 199–206. 10.1179/2045772313Y.0000000179 24621048 PMC4397202

[B139] Nishiumi S. Miyamoto S. Kawabata K. Ohnishi K. Mukai R. Murakami A. (2011). Dietary Flavonoids as Cancer-Preventive and Therapeutic Biofactors. Front. Biosci. (Schol Ed. 3, 1332–1362. 10.2741/229 21622274

[B140] Niu C. Aisa H. A. (2017). Upregulation of Melanogenesis and Tyrosinase Activity: Potential Agents for Vitiligo. Molecules 22 (8). 10.3390/molecules22081303 PMC6152334 28777326

[B141] O'Donovan P. Perrett C. M. Zhang X. Montaner B. Xu Y. Z. Harwood C. A. (2005). Azathioprine and UVA Light Generate Mutagenic Oxidative DNA Damage. Science 309 (5742), 1871–1874. 10.1126/science.1114233 16166520 PMC2426755

[B142] Oh J. H. Kim J. Karadeniz F. Kim H. R. Park S. Y. Seo Y. (2021). Santamarine Shows Anti-photoaging Properties via Inhibition of MAPK/AP-1 and Stimulation of TGF-β/Smad Signaling in UVA-Irradiated HDFs. Molecules 26 (12). 10.3390/molecules26123585 PMC8230857 34208202

[B143] Onkoksoong T. Jeayeng S. Poungvarin N. Limsaengurai S. Thamsermsang O. Tripatara P. (2018). Thai Herbal Antipyretic 22 Formula (APF22) Inhibits UVA-Mediated Melanogenesis through Activation of Nrf2-Regulated Antioxidant Defense. Phytother Res. 32 (8), 1546–1554. 10.1002/ptr.6083 29672960

[B144] Panche A. N. Diwan A. D. Chandra S. R. (2016). Flavonoids: an Overview. J. Nutr. Sci. 5, e47. 10.1017/jns.2016.41 28620474 PMC5465813

[B145] Pandey K. B. Rizvi S. I. (2009). Plant Polyphenols as Dietary Antioxidants in Human Health and Disease. Oxid Med. Cell Longev 2 (5), 270–278. 10.4161/oxim.2.5.9498 20716914 PMC2835915

[B146] Panich U. Pluemsamran T. Tangsupa-a-nan V. Wattanarangsan J. Phadungrakwittaya R. Akarasereenont P. (2013). Protective Effect of AVS073, a Polyherbal Formula, against UVA-Induced Melanogenesis through a Redox Mechanism Involving Glutathione-Related Antioxidant Defense. BMC Complement. Altern. Med. 13, 159. 10.1186/1472-6882-13-159 23826868 PMC3706233

[B147] Park C. Lee H. Noh J. S. Jin C. Y. Kim G. Y. Hyun J. W. (2020). Hemistepsin A Protects Human Keratinocytes against Hydrogen Peroxide-Induced Oxidative Stress through Activation of the Nrf2/HO-1 Signaling Pathway. Arch. Biochem. Biophys. 691, 108512. 10.1016/j.abb.2020.108512 32712291

[B148] Park C. Park J. Kim W.-J. Kim W. Cheong H. Kim S.-J. (2021). Malonic Acid Isolated from Pinus Densiflora Inhibits UVB-Induced Oxidative Stress and Inflammation in HaCaT Keratinocytes. Polymers 13 (5), 816. 10.3390/polym13050816 33799974 PMC7961482

[B149] Parrado C. Mascaraque M. Gilaberte Y. Juarranz A. Gonzalez S. (2016). Fernblock (Polypodium Leucotomos Extract): Molecular Mechanisms and Pleiotropic Effects in Light-Related Skin Conditions, Photoaging and Skin Cancers, a Review. Int. J. Mol. Sci. 17 (7). 10.3390/ijms17071026 PMC4964402 27367679

[B150] Pastore S. Lulli D. Pascarella A. Maurelli R. Dellambra E. Potapovich A. (2012). Resveratrol Enhances Solar UV-Induced Responses in normal Human Epidermal Keratinocytes. Photochem. Photobiol. 88 (6), 1522–1530. 10.1111/j.1751-1097.2012.01195.x 22762504

[B151] Pittayapruek P. Meephansan J. Prapapan O. Komine M. Ohtsuki M. (2016). Role of Matrix Metalloproteinases in Photoaging and Photocarcinogenesis. Int. J. Mol. Sci. 17 (6). 10.3390/ijms17060868 PMC4926402 27271600

[B152] Pramanik R. Qi X. Borowicz S. Choubey D. Schultz R. M. Han J. (2003). p38 Isoforms Have Opposite Effects on AP-1-dependent Transcription through Regulation of C-Jun. The Determinant Roles of the Isoforms in the P38 MAPK Signal Specificity. J. Biol. Chem. 278 (7), 4831–4839. 10.1074/jbc.M207732200 12475989

[B153] Quan T. Qin Z. Xia W. Shao Y. Voorhees J. J. Fisher G. J. (2009). Matrix-degrading Metalloproteinases in Photoaging. J. Investig. Dermatol. Symp. Proc. 14 (1), 20–24. 10.1038/jidsymp.2009.8 PMC2909639 19675548

[B154] Rijken F. Bruijnzeel-Koomen C. A. (2011). Photoaged Skin: the Role of Neutrophils, Preventive Measures, and Potential Pharmacological Targets. Clin. Pharmacol. Ther. 89 (1), 120–124. 10.1038/clpt.2010.221 21107312

[B155] Rinnerthaler M. Bischof J. Streubel M. K. Trost A. Richter K. (2015). Oxidative Stress in Aging Human Skin. Biomolecules 5 (2), 545–589. 10.3390/biom5020545 25906193 PMC4496685

[B156] Rittié L. Fisher G. J. (2002). UV-light-induced Signal Cascades and Skin Aging. Ageing Res. Rev. 1 (4), 705–720. 10.1016/s1568-1637(02)00024-7 12208239

[B157] Rodríguez-Luna A. Ávila-Román J. González-Rodríguez M. L. Cózar M. J. Rabasco A. M. Motilva V. (2018). Fucoxanthin-Containing Cream Prevents Epidermal Hyperplasia and UVB-Induced Skin Erythema in Mice. Mar. Drugs 16 (10). 10.3390/md16100378 PMC6212948 30308980

[B158] Rodríguez-Luna A. Ávila-Román J. Oliveira H. Motilva V. Talero E. (2019). Fucoxanthin and Rosmarinic Acid Combination Has Anti-inflammatory Effects through Regulation of NLRP3 Inflammasome in UVB-Exposed HaCaT Keratinocytes. Mar. Drugs 17 (8). 10.3390/md17080451 PMC6722862 31374828

[B159] Rojo de la Vega M. Krajisnik A. Zhang D. D. Wondrak G. T. (2017). Targeting NRF2 for Improved Skin Barrier Function and Photoprotection: Focus on the Achiote-Derived Apocarotenoid Bixin. Nutrients 9 (12). 10.3390/nu9121371 PMC5748821 29258247

[B160] Ryšavá A. Čížková K. Franková J. Roubalová L. Ulrichová J. Vostálová J. (2020). Effect of UVA Radiation on the Nrf2 Signalling Pathway in Human Skin Cells. J. Photochem. Photobiol. B: Biol. 209, 111948. 10.1016/j.jphotobiol.2020.111948 32679512

[B161] Ryšavá A. Vostálová J. Rajnochová Svobodová A. (2021). Effect of Ultraviolet Radiation on the Nrf2 Signaling Pathway in Skin Cells. Int. J. Radiat. Biol. 97 (10), 1383–1403. 10.1080/09553002.2021.1962566 34338112

[B162] Ryu J. H. Seok J. K. An S. M. Baek J. H. Koh J. S. Boo Y. C. (2015). A Study of the Human Skin-Whitening Effects of Resveratryl Triacetate. Arch. Dermatol. Res. 307 (3), 239–247. 10.1007/s00403-015-1556-0 25750159

[B163] Saha S. Buttari B. Panieri E. Profumo E. Saso L. (2020). An Overview of Nrf2 Signaling Pathway and its Role in Inflammation. Molecules 25 (22). 10.3390/molecules25225474 PMC7700122 33238435

[B164] Sangaraju R. Alavala S. Nalban N. Jerald M. K. Sistla R. (2021). Galangin Ameliorates Imiquimod-Induced Psoriasis-like Skin Inflammation in BALB/c Mice via Down Regulating NF-κB and Activation of Nrf2 Signaling Pathways. Int. Immunopharmacol 96, 107754. 10.1016/j.intimp.2021.107754 34162135

[B165] Saw C. L. Huang M. T. Liu Y. Khor T. O. Conney A. H. Kong A. N. (2011). Impact of Nrf2 on UVB-Induced Skin Inflammation/photoprotection and Photoprotective Effect of Sulforaphane. Mol. Carcinog 50 (6), 479–486. 10.1002/mc.20725 21557329

[B166] Schäfer M. Farwanah H. Willrodt A. H. Huebner A. J. Sandhoff K. Roop D. (2012). Nrf2 Links Epidermal Barrier Function with Antioxidant Defense. EMBO Mol. Med. 4 (5), 364–379. 10.1002/emmm.201200219 22383093 PMC3403295

[B167] Schäfer M. Dütsch S. auf dem Keller U. Navid F. Schwarz A. Johnson D. A. (2010). auf dem Keller, UNrf2 establishes a glutathione-mediated gradient of UVB cytoprotection in the epidermis. Genes Dev. 24 (10), 1045–1058. 10.1101/gad.568810 20478997 PMC2867209

[B168] Schmitz S. Thomas P. D. Allen T. M. Poznansky M. J. Jimbow K. (1995). Dual Role of Melanins and Melanin Precursors as Photoprotective and Phototoxic Agents: Inhibition of Ultraviolet Radiation-Induced Lipid Peroxidation. Photochem. Photobiol. 61 (6), 650–655. 10.1111/j.1751-1097.1995.tb09883.x 7568412

[B169] Searle T. Al-Niaimi F. Ali F. R. (2020). The Top 10 Cosmeceuticals for Facial Hyperpigmentation. Dermatol. Ther. 33 (6), e14095. 10.1111/dth.14095 32720446

[B170] Serini S. Guarino R. Ottes Vasconcelos R. Celleno L. Calviello G. (2020). The Combination of Sulforaphane and Fernblock® XP Improves Individual Beneficial Effects in Normal and Neoplastic Human Skin Cell Lines. Nutrients 12 (6). 10.3390/nu12061608 PMC7353001 32486135

[B171] Shao Y. Dang M. Lin Y. Xue F. (2019). Evaluation of Wound Healing Activity of Plumbagin in Diabetic Rats. Life Sci. 231, 116422. 10.1016/j.lfs.2019.04.048 31059689

[B172] Sheen Y. S. Huang H. Y. Liao Y. H. (2021). The Efficacy and Safety of an Antiaging Topical Serum Containing Hesperetin and Sodium Cyclic Lysophosphatidic Acid: A Single‐center Clinical Trial. J. Cosmet. Dermatol. 20, 3960–3967. 10.1111/jocd.14063 33690913

[B173] Shin J. M. Kim M. Y. Sohn K. C. Jung S. Y. Lee H. E. Lim J. W. (2014). Nrf2 Negatively Regulates Melanogenesis by Modulating PI3K/Akt Signaling. PLoS One 9 (4), e96035. 10.1371/journal.pone.0096035 24763530 PMC3999113

[B174] Shin J. W. Chun K. S. Kim D. H. Kim S. J. Kim S. H. Cho N. C. (2020). Curcumin Induces Stabilization of Nrf2 Protein through Keap1 Cysteine Modification. Biochem. Pharmacol. 173, 113820. 10.1016/j.bcp.2020.113820 31972171

[B175] Shirasugi I. Kamada M. Matsui T. Sakakibara Y. Liu M. C. Suiko M. (2010). Sulforaphane Inhibited Melanin Synthesis by Regulating Tyrosinase Gene Expression in B16 Mouse Melanoma Cells. Biosci. Biotechnol. Biochem. 74 (3), 579–582. 10.1271/bbb.90778 20208349

[B176] Shoji T. Masumoto S. Moriichi N. Ohtake Y. Kanda T. (2020). Administration of Apple Polyphenol Supplements for Skin Conditions in Healthy Women: A Randomized, Double-Blind, Placebo-Controlled Clinical Trial. Nutrients 12 (4). 10.3390/nu12041071 PMC7231294 32294883

[B177] Shroff H. Diedrichs P. C. Craddock N. (2017). Skin Color, Cultural Capital, and Beauty Products: An Investigation of the Use of Skin Fairness Products in Mumbai, India. Front. Public Health 5, 365. 10.3389/fpubh.2017.00365 29410952 PMC5787082

[B178] Silva S. A. M. E. Michniak-Kohn B. Leonardi G. R. (2017). An Overview about Oxidation in Clinical Practice of Skin Aging. Bras Dermatol. 92 (3), 367–374. 10.1590/abd1806-4841.20175481 PMC5514578 29186250

[B179] Silvers A. L. Bachelor M. A. Bowden G. T. (2003). The Role of JNK and P38 MAPK Activities in UVA-Induced Signaling Pathways Leading to AP-1 Activation and C-Fos Expression. Neoplasia 5 (4), 319–329. 10.1016/S1476-5586(03)80025-8 14511403 PMC1502419

[B180] Sirerol J. A. Feddi F. Mena S. Rodriguez M. L. Sirera P. Aupí M. (2015). Topical Treatment with Pterostilbene, a Natural Phytoalexin, Effectively Protects Hairless Mice against UVB Radiation-Induced Skin Damage and Carcinogenesis. Free Radic. Biol. Med. 85, 1–11. 10.1016/j.freeradbiomed.2015.03.027 25845487

[B181] Slominski A. Tobin D. J. Shibahara S. Wortsman J. (2004). Melanin Pigmentation in Mammalian Skin and its Hormonal Regulation. Physiol. Rev. 84 (4), 1155–1228. 10.1152/physrev.00044.2003 15383650

[B182] Slominski A. Wortsman J. Luger T. Paus R. Solomon S. (2000). Corticotropin Releasing Hormone and Proopiomelanocortin Involvement in the Cutaneous Response to Stress. Physiol. Rev. 80 (3), 979–1020. 10.1152/physrev.2000.80.3.979 10893429

[B183] Smalley K. Eisen T. (2000). The Involvement of P38 Mitogen-Activated Protein Kinase in the Alpha-Melanocyte Stimulating Hormone (Alpha-MSH)-Induced Melanogenic and Anti-proliferative Effects in B16 Murine Melanoma Cells. FEBS Lett. 476 (3), 198–202. 10.1016/s0014-5793(00)01726-9 10913613

[B184] Sporn M. B. Liby K. T. (2012). NRF2 and Cancer: the Good, the Bad and the Importance of Context. Nat. Rev. Cancer 12 (8), 564–571. 10.1038/nrc3278 22810811 PMC3836441

[B185] Staurengo-Ferrari L. Badaro-Garcia S. Hohmann M. S. N. Manchope M. F. Zaninelli T. H. Casagrande R. (2018). Contribution of Nrf2 Modulation to the Mechanism of Action of Analgesic and Anti-inflammatory Drugs in Pre-clinical and Clinical Stages. Front. Pharmacol. 9, 1536. 10.3389/fphar.2018.01536 30687097 PMC6337248

[B186] Su T. R. Lin J. J. Tsai C. C. Huang T. K. Yang Z. Y. Wu M. O. (2013). Inhibition of Melanogenesis by Gallic Acid: Possible Involvement of the PI3K/Akt, MEK/ERK and Wnt/β-Catenin Signaling Pathways in B16F10 Cells. Int. J. Mol. Sci. 14 (10), 20443–20458. 10.3390/ijms141020443 24129178 PMC3821624

[B187] Sun Z. Park S. Y. Hwang E. Park B. Seo S. A. Cho J. G. (2016). Dietary Foeniculum Vulgare Mill Extract Attenuated UVB Irradiation-Induced Skin Photoaging by Activating of Nrf2 and Inhibiting MAPK Pathways. Phytomedicine 23 (12), 1273–1284. 10.1016/j.phymed.2016.06.008 27765346

[B188] Süntar I. Çetinkaya S. Panieri E. Saha S. Buttari B. Profumo E. (2021). Regulatory Role of Nrf2 Signaling Pathway in Wound Healing Process. Molecules 26 (9). 10.3390/molecules26092424 PMC8122529 33919399

[B189] Surh Y. J. (2003). Cancer Chemoprevention with Dietary Phytochemicals. Nat. Rev. Cancer 3 (10), 768–780. 10.1038/nrc1189 14570043

[B190] Surh Y. J. Na H. K. (2008). NF-kappaB and Nrf2 as Prime Molecular Targets for Chemoprevention and Cytoprotection with Anti-inflammatory and Antioxidant Phytochemicals. Genes Nutr. 2 (4), 313–317. 10.1007/s12263-007-0063-0 18850223 PMC2478481

[B191] Suschek C. V. Briviba K. Bruch-Gerharz D. Sies H. Kröncke K. D. Kolb-Bachofen V. (2001). Even after UVA-Exposure Will Nitric Oxide Protect Cells from Reactive Oxygen Intermediate-Mediated Apoptosis and Necrosis. Cell Death Differ 8 (5), 515–527. 10.1038/sj.cdd.4400839 11423912

[B192] Suzuki T. Muramatsu A. Saito R. Iso T. Shibata T. Kuwata K. (2019). Molecular Mechanism of Cellular Oxidative Stress Sensing by Keap1. Cell Rep 28 (3), 746–e4. 10.1016/j.celrep.2019.06.047 31315052

[B193] Swiader A. Camaré C. Guerby P. Salvayre R. Negre-Salvayre A. (2021). 4-Hydroxynonenal Contributes to Fibroblast Senescence in Skin Photoaging Evoked by UV-A Radiation. Antioxidants (Basel) 10 (3). 10.3390/antiox10030365 PMC7997366 33670907

[B194] Taguchi K. Yamamoto M. (2020). The KEAP1-NRF2 System as a Molecular Target of Cancer Treatment. Cancers (Basel) 13 (1). 10.3390/cancers13010046 PMC7795874 33375248

[B195] Takei K. Hashimoto-Hachiya A. Takahara M. Tsuji G. Nakahara T. Furue M. (2015). Cynaropicrin Attenuates UVB-Induced Oxidative Stress via the AhR-Nrf2-Nqo1 Pathway. Toxicol. Lett. 234 (2), 74–80. 10.1016/j.toxlet.2015.02.007 25680693

[B196] Tan Y. Ichikawa T. Li J. Si Q. Yang H. Chen X. (2011). Diabetic Downregulation of Nrf2 Activity via ERK Contributes to Oxidative Stress-Induced Insulin Resistance in Cardiac Cells *In Vitro* and *In Vivo* . Diabetes 60 (2), 625–633. 10.2337/db10-1164 21270272 PMC3028364

[B197] Tran T. T. Schulman J. Fisher D. E. (2008). UV and Pigmentation: Molecular Mechanisms and Social Controversies. Pigment Cell Melanoma Res 21 (5), 509–516. 10.1111/j.1755-148X.2008.00498.x 18821855 PMC2733367

[B198] Truong V. L. Bak M. J. Jun M. Kong A. N. Ho C. T. Jeong W. S. (2014). Antioxidant Defense and Hepatoprotection by Procyanidins from almond (Prunus Amygdalus) Skins. J. Agric. Food Chem. 62 (34), 8668–8678. 10.1021/jf5027247 25119859

[B199] Uto T. Hou D. X. Morinaga O. Shoyama Y. (2012). Molecular Mechanisms Underlying Anti-inflammatory Actions of 6-(Methylsulfinyl)hexyl Isothiocyanate Derived from Wasabi (Wasabia Japonica). Adv. Pharmacol. Sci. 2012, 614046. 10.1155/2012/614046 22927840 PMC3426159

[B200] Vacek J. Vostalova J. Papouskova B. Skarupova D. Kos M. Kabelac M. (2021). Antioxidant Function of Phytocannabinoids: Molecular Basis of Their Stability and Cytoprotective Properties under UV-Irradiation. Free Radic. Biol. Med. 164, 258–270. 10.1016/j.freeradbiomed.2021.01.012 33453360

[B201] Vomund S. Schäfer A. Parnham M. J. Brüne B. von Knethen A. (2017). Nrf2, the Master Regulator of Anti-Oxidative Responses. Int. J. Mol. Sci. 18 (12). 10.3390/ijms18122772 PMC5751370 29261130

[B202] Wagner A. E. Boesch-Saadatmandi C. Dose J. Schultheiss G. Rimbach G. (2012). Anti-inflammatory Potential of Allyl-Isothiocyanate-Rrole of Nrf2, NF-(κ) B and microRNA-155. J. Cell Mol Med 16 (4), 836–843. 10.1111/j.1582-4934.2011.01367.x 21692985 PMC3822852

[B203] Wang P. W. Cheng Y. C. Hung Y. C. Lee C. H. Fang J. Y. Li W. T. (2019). Red Raspberry Extract Protects the Skin against UVB-Induced Damage with Antioxidative and Anti-inflammatory Properties. Oxid Med. Cell Longev 2019, 9529676. 10.1155/2019/9529676 30723535 PMC6339709

[B204] Wang S. Wu Y. Y. Wang X. Shen P. Jia Q. Yu S. (2020). Lycopene Prevents Carcinogen-Induced Cutaneous Tumor by Enhancing Activation of the Nrf2 Pathway through P62-Triggered Autophagic Keap1 Degradation. Aging (Albany NY) 12 (9), 8167–8190. 10.18632/aging.103132 32365333 PMC7244072

[B205] Wang Y. Hao M. M. Sun Y. Wang L. F. Wang H. Zhang Y. J. (2018). Synergistic Promotion on Tyrosinase Inhibition by Antioxidants. Molecules 23 (1). 10.3390/molecules23010106 PMC6017046 29300356

[B206] Watanabe H. Shimizu T. Nishihira J. Abe R. Nakayama T. Taniguchi M. (2004). Ultraviolet A-Induced Production of Matrix Metalloproteinase-1 Is Mediated by Macrophage Migration Inhibitory Factor (MIF) in Human Dermal Fibroblasts. J. Biol. Chem. 279 (3), 1676–1683. 10.1074/jbc.M303650200 14581488

[B207] Watson M. Holman D. M. Maguire-Eisen M. (2016). Ultraviolet Radiation Exposure and its Impact on Skin Cancer Risk. Semin. Oncol. Nurs. 32 (3), 241–254. 10.1016/j.soncn.2016.05.005 27539279 PMC5036351

[B208] Whitmarsh A. J. (2007). Regulation of Gene Transcription by Mitogen-Activated Protein Kinase Signaling Pathways. Biochim. Biophys. Acta 1773 (8), 1285–1298. 10.1016/j.bbamcr.2006.11.011 17196680

[B209] Wikramanayake T. C. Stojadinovic O. Tomic-Canic M. (2014). Epidermal Differentiation in Barrier Maintenance and Wound Healing. Adv. Wound Care (New Rochelle) 3 (3), 272–280. 10.1089/wound.2013.0503 24669361 PMC3955965

[B210] Wu M. Y. Hung S. K. Fu S. L. (2011). Immunosuppressive Effects of Fisetin in Ovalbumin-Induced Asthma through Inhibition of NF-κB Activity. J. Agric. Food Chem. 59 (19), 10496–10504. 10.1021/jf202756f 21899296

[B211] Wu P. Y. Lyu J. L. Liu Y. J. Chien T. Y. Hsu H. C. Wen K. C. (2017). Fisetin Regulates Nrf2 Expression and the Inflammation-Related Signaling Pathway to Prevent UVB-Induced Skin Damage in Hairless Mice. Int. J. Mol. Sci. 18 (10). 10.3390/ijms18102118 PMC5666800 28994699

[B212] Wu P. Y. You Y. J. Liu Y. J. Hou C. W. Wu C. S. Wen K. C. (2018). Sesamol Inhibited Melanogenesis by Regulating Melanin-Related Signal Transduction in B16F10 Cells. Int. J. Mol. Sci. 19 (4). 10.3390/ijms19041108 PMC5979541 29642438

[B213] Wu W. Peng G. Yang F. Zhang Y. Mu Z. Han X. (2019). Sulforaphane Has a Therapeutic Effect in an Atopic Dermatitis Murine Model and Activates the Nrf2/HO-1 axis. Mol. Med. Rep. 20 (2), 1761–1771. 10.3892/mmr.2019.10405 31257541 PMC6625393

[B214] Xian D. Xiong X. Xu J. Xian L. Lei Q. Song J. (2019). Nrf2 Overexpression for the Protective Effect of Skin-Derived Precursors against UV-Induced Damage: Evidence from a Three-Dimensional Skin Model. Oxid Med. Cell Longev 2019, 7021428. 10.1155/2019/7021428 31737172 PMC6815583

[B215] Yamamoto T. Suzuki T. Kobayashi A. Wakabayashi J. Maher J. Motohashi H. (2008). Physiological Significance of Reactive Cysteine Residues of Keap1 in Determining Nrf2 Activity. Mol. Cell Biol 28 (8), 2758–2770. 10.1128/MCB.01704-07 18268004 PMC2293100

[B216] Yamashita Y. Ikeda T. Matsuda M. Maji D. Hoshino T. Mizushima T. (2012). Purification and Characterization of HSP-Inducers from Eupatorium Lindleyanum. Biochem. Pharmacol. 83 (7), 909–922. 10.1016/j.bcp.2011.12.040 22245466

[B217] Yang H. L. Lin C. P. Vudhya Gowrisankar Y. Huang P. J. Chang W. L. Shrestha S. (2021). The Anti-melanogenic Effects of Ellagic Acid through Induction of Autophagy in Melanocytes and Suppression of UVA-Activated α-MSH Pathways via Nrf2 Activation in Keratinocytes. Biochem. Pharmacol. 185, 114454. 10.1016/j.bcp.2021.114454 33545118

[B218] Yang K. Kim S. Y. Park J. H. Ahn W. G. Jung S. H. Oh D. (2020). Topical Application of Phlorotannins from Brown Seaweed Mitigates Radiation Dermatitis in a Mouse Model. Mar. Drugs 18 (8). 10.3390/md18080377 PMC7460453 32707897

[B219] Zhao P. Alam M. B. Lee S. H. (2018). Protection of UVB-Induced Photoaging by Fuzhuan-Brick Tea Aqueous Extract via MAPKs/Nrf2-Mediated Down-Regulation of MMP-1. Nutrients 11 (1). 10.3390/nu11010060 PMC6357030 30597920

[B220] Zhou X. Ruan Q. Ye Z. Chu Z. Xi M. Li M. (2021). Resveratrol Accelerates Wound Healing by Attenuating Oxidative Stress-Induced Impairment of Cell Proliferation and Migration. Burns 47 (1), 133–139. 10.1016/j.burns.2020.10.016 33288327

